# A phase I study of anti‐BCMA CAR T cell therapy in relapsed/refractory multiple myeloma and plasma cell leukemia

**DOI:** 10.1002/ctm2.346

**Published:** 2021-03-09

**Authors:** Chunrui Li, Wenyue Cao, Yimei Que, Qiuxiang Wang, Yi Xiao, Chaojiang Gu, Di Wang, Jue Wang, Lijun Jiang, Hao Xu, Jinhuan Xu, Xiaoxi Zhou, Zhenya Hong, Na Wang, Liang Huang, Shangkun Zhang, Liting Chen, Xia Mao, Min Xiao, Wei Zhang, Li Meng, Yang Cao, Tongcun Zhang, Jian Li, Jianfeng Zhou

**Affiliations:** ^1^ Department of Hematology, Tongji Hospital, Tongji Medical College Huazhong University of Science and Technology Wuhan Hubei China; ^2^ Immunotherapy Research Center for Hematologic Diseases of Hubei Province Wuhan Hubei China; ^3^ Wuhan No.1 Hospital Wuhan Hubei China; ^4^ College of Life Science and Health Wuhan University of Science and Technology Wuhan Hubei China; ^5^ Wuhan Bio‐Raid Biotechnology Co., Ltd. Wuhan Hubei China; ^6^ Department of Hematology Peking Union Medical College Hospital Chinese Academy of Medical Sciences and Peking Union Medical College Beijing China

**Keywords:** anti‐BCMA CAR T cell, multiple myeloma, plasma cell leukemia, relapsed/refractory

## Abstract

**Background:**

Relapsed/refractory (R/R) multiple myeloma (MM) patients and primary plasma cell leukemia (PCL) have an unfavorable prognosis and no effective treatment. This study was designed to assess the safety and preliminary efficacy of a novel anti‐B‐cell maturation antigen (BCMA) chimeric antigen receptor (CAR) T cell in R/R MM and PCL.

**Methods:**

Between February 22, 2017, and June 25, 2018, 28 R/R and two R/R primary PCL patients received a median dose of 11.2 × 10^6^ CAR+ cells/kg. The subjects were refractory to a proteasome inhibitor and/or an immunomodulatory agent. Fludarabine and cyclophosphamide were given as lymphodepletion chemotherapy.

**Results:**

Results for these 30 consecutive patients who received an anti‐BCMA CAR T cell infusion are reported. The patients had received a median of four prior lines of therapy. A total of 44 different types of adverse events were recorded, and hematologic toxic effects were the most common events of any grade during treatment. Hematologic toxic effects were also the most common events of grade 3 or higher. A total of 29 patients (96.7%) had cytokine release syndrome, which was of grade 1 or 2 in 24 patients (80%) and grade 3 in five patients (16.7%). Neurologic toxic effects only occurred in one patient (3.3%) and were of grade 1. The objective response rate was 90%, and the complete response rate was 43.3%. With a median follow‐up of 12.6 months, the median progression‐free survival (PFS) and overall survival were 5.2 months and 14.0 months. One of the two primary PCL achieved a complete response with a PFS of 307 days. The other patients achieved a very good partial response with a PFS of 117 days.

**Conclusions:**

Anti‐BCMA CAR T cell treatment is safe and highly active in R/R multiple myeloma.

## Graphical abstract

1. We report the efficacy and safety of the infusion of anti‐BCMA CAR T cell treatment in with relapsed/refractory malignant plasma cell disease.
2. Anti‐BCMA CAR T cell therapy exerted better safety and preliminary efficacy in relapsed/refractory multiple myeloma.
3. Relapsed/refractory primary plasma cell leukemia may benefit from CAR T Cell treatment, although the duration of response is short.

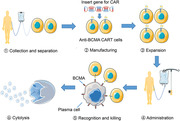

## 1 INTRODUCTION

Despite recent improvements in treatment, multiple myeloma (MM) remains an almost incurable disease associated with high morbidity and mortality. Recent advances in MM treatment strategies, particularly the clinical application of novel immunomodulatory drugs, proteasome inhibitors, and monoclonal antibodies, have largely improved the response rate, progression‐free survival (PFS), and overall survival (OS) in relapsed MM patients.^1^, ^2^, ^3^, ^4^ However, since drug‐resistant clones emerge and evolve unavoidably and continuously,^5^ almost all patients eventually have a relapse with worse survival outcomes.^6^, ^7^, ^8^ Thus, more efficacious therapies and novel strategies are still urgently needed.

Plasma cell leukemia (PCL) is a particularly aggressive variety of plasma cell disorders and is often occurring in patients with relapsed/refractory (R/R) MM. Compared with MM, the clinical course of PCL is more aggressive with an unfavorable prognosis, and the management of PCL remains challenging.^9^, ^10^ Currently, data on PCL patients have not been included in most of the clinical studies on MM, and this under‐representation is an unmet need.

Clinical trials employing chimeric antigen receptor (CAR) T cells to treat MM are ongoing and have generated some promising results.^11^, ^12^, ^13^, ^14^, ^15^ B‐cell maturation antigen (BCMA), a member of the tumor necrosis factor superfamily proteins, is specifically highly expressed on malignant and normal plasma cells and some mature B cells, making it a potential target for MM.^16^, ^17^, ^18^, ^19^

We developed a novel BCMA‐targeted CAR T cells therapy, and now report results from our phase I clinical trial evaluating anti‐BCMA CAR T Cell in R/R MM patients and primary PCL, which, to our knowledge, represents the first report involving CAR T cell treatment for primary PCL.

## 2 MATERIALS AND METHODS

### 2.1 Study conduct and patients

This study was conducted in compliance with the Declaration of Helsinki. The study protocol was approved by the institutional review board of Tongji Hospital, Tongji Medical College, Huazhong University of Science and Technology and registered with the Chinese Clinical Trial Registry (http://www.chictr.org.cn. Number, ChiCTR‐OPC‐16009113). Written informed consent was obtained from each participant, in compliance with the Declaration of Helsinki. Eligible patients were subjects with BCMA+ R/R MM and PCL. They were diagnosed according to the International Myeloma Working Group (IMWG) updated criteria.^19^ Good performance status, necessarily adequate major organ function, measurable disease, and a life expectancy of 3 months or more were required for eligibility.

**FIGURE 1:**
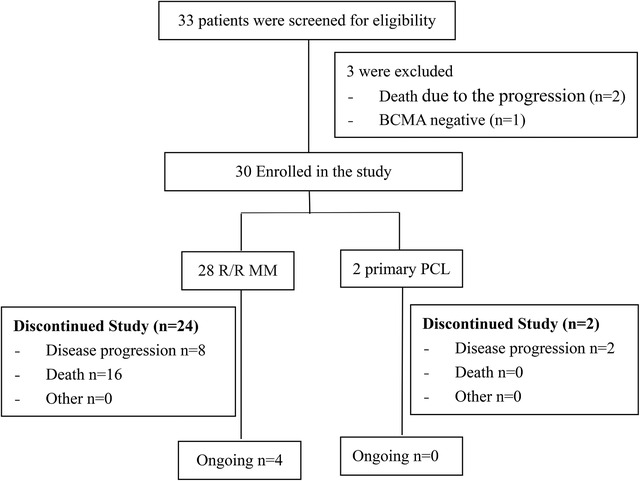
Consort diagram

In total, 33 patients were screened for eligibility, and three were excluded for death or BCMA negative. Among those excluded from the trial, two patients died due to the progression before CAR T infusions, and one patient was excluded for BCMA negative (Figure 1). No patient had received bridge chemotherapy before CAR T infusions. All eligible patients underwent leukapheresis. A lymphodepleting chemotherapy with cyclophosphamide 20 mg/kg and fludarabine 25 mg/m^2^ daily on days 4–2 was administered before infusion of anti‐BCMA CAR T cells on day 0. Related laboratory and imaging evaluations were performed for toxicity and response assessment according to the study procedures. Anti‐BCMA CAR T cells were generally administered on days 0–2. Subjects may receive 5.0–30.0 × 10^6^ CAR+ cells/kg (a deviation within ± 20% also acceptable) according to the number of anti‐BCMA CAR T cells manufactured. Subjects with a number of CAR+ T cells manufactured below 5.0 × 10^6^ CAR+ cells/kg were excluded from this study. Further details regarding patient treatment and study procedures are described in the Supplemental Methods.

**HIGHLIGHTS**

1

1. Anti‐BCMA CAR T cell therapy exerted better safety and preliminary efficacy in relapsed/refractoryR/R multiple myeloma.
2. Relapsed/refractoryR/R primary plasma cell leukemia patients may benefit from CAR T Cell cell treatment, although the duration of response is short.
3. It is necessary to clarify the mechanism of relapse after CAR T cell treatment.

### 2.2 Preparation of BCMA CAR T cells

The lentiviral vector containing a BCMA CAR with a murine anti‐BCMA single‐chain variable fragment, a CD8a hinge, the CD28 costimulatory molecule, and the intracellular signaling domain of CD3ζ was produced in Lenti‐X 293T cells (Takara Bio Inc, Beijing, China). Autologous peripheral blood mononuclear cells (PBMC, range = 80–100 ml of blood) were isolated from each patient by low‐density centrifugation on lymphocyte separation medium (GE Healthcare, Chicago, IL, USA), and T lymphocytes were separated from these using MACS human CD3 microbeads (Miltenyi Biotec GmbH, Germany) following the manufacturer's instructions. After activation overnight with Dynabeads Human T‐Activator CD3/CD28 (Invitrogen), T cells were transduced with lentivirus at a multiplicity of infection (MOI) ranging from 3 to 5.

**FIGURE 2:**
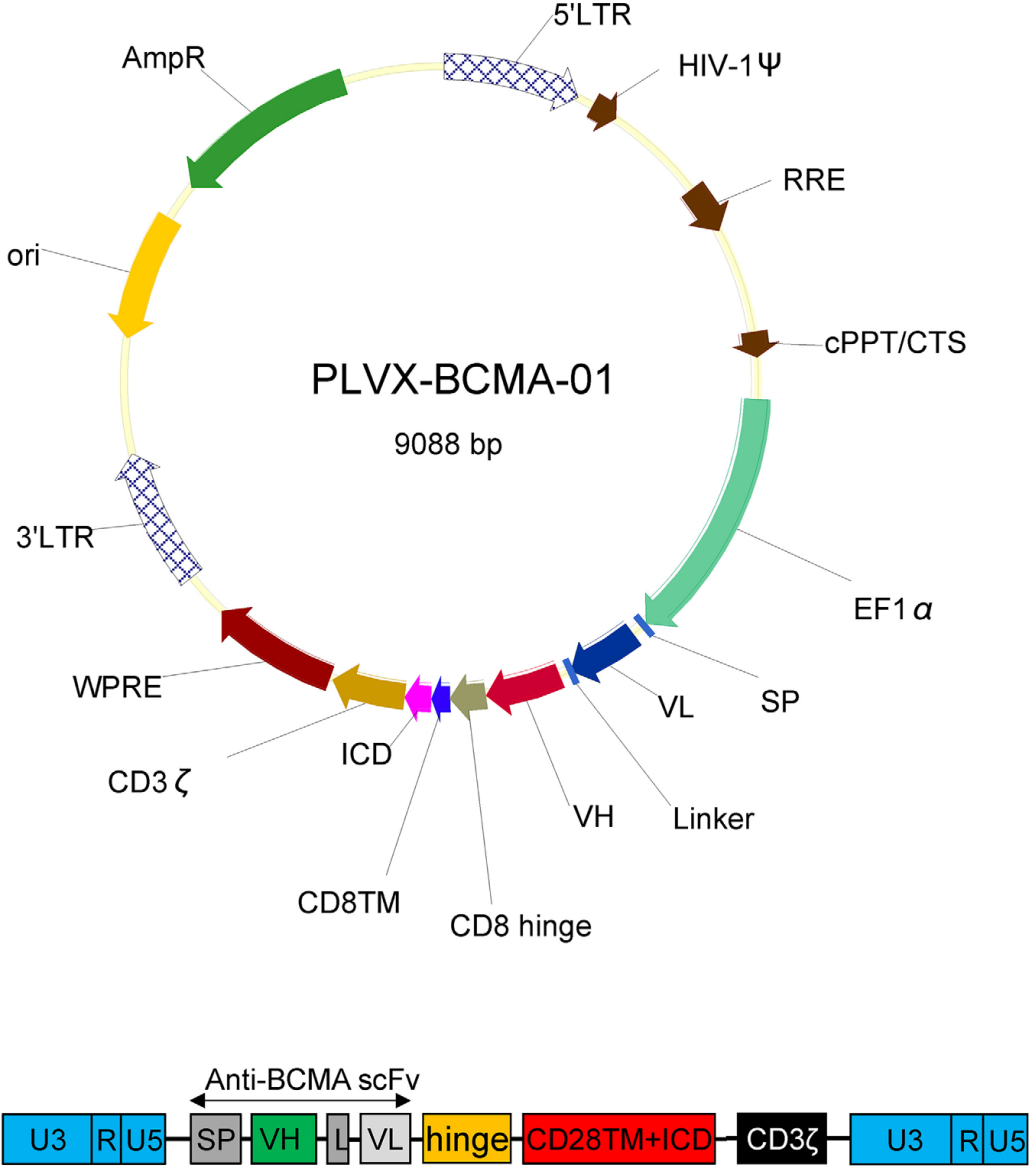
Schematic diagrams of lentiviral vector and CAR constructs. Upper, schematic diagram of lentiviral vector. Lower, schematic diagram of anti‐BCMA CAR. The second‐generation CAR utilized in this trial was composed of a single‐chain variable fragment (scFv) derived from a murine monoclonal antibody against human BCMA, the costimulatory domain from CD28, and the CD3ζ chain as an activation domain Abbreviations: L, linker; SP, signal peptide; VH, variable H chain; VL, variable L chain.

Transduction efficiencies were monitored by flow cytometric analysis after 3 days and were within an appropriate range (median = 0.46%, interquartile range (IQR) = [0.39%, 0.54%]). All the CD3+ T cells were cultured using CTS OpTmizer medium (Gibco) with additional rhIL‐2 (PeproTech), HEPES, and L‐Glutamine (Gibco). The median time of CAR T‐cells culture before infusion was 16 days (range, 12–25 days). The cytotoxicity of CAR T‐cells was detected by the Calcein release assay, and the CAR‐Ts showed adequate tumor‐killing effectiveness in vitro (median = 0.57%, IQR = [0.34%, 0.81%]). The detailed data on patients’ CAR T manufacturing information, including transfection efficiency, duration of culturing, cytolysis rates, were provided in Table S1 in the Supplementary Appendix. The schematic diagrams of the lentiviral vector and CAR constructs were shown in Figure 2.

### 2.3 End points and assessments

The primary endpoint was to evaluate the safety, including the incidence of adverse events (AEs), severe AEs (SAE), and cytokine release syndrome (CRS). Secondary endpoints were preliminary efficacy, OS, and PFS. According to the IMWG consensus criteria, clinical response and disease progression were evaluated every month for the first 6 months and then every 2 months till 1 year after anti‐BCMA CAR T cells infusion.^20^ The CRS was graded according to the criteria of Lee et al.^21^ CAR T cell‐related encephalopathy syndrome and other AEs were evaluated according to the National Cancer Institute Common Terminology Criteria for AEs V4.03.^22^ Minimal residual disease (MRD) in bone marrow was measured by multiparameter flow cytometry (MFC) with a minimum cutoff of 10^−4^ nucleated cells. The definition of extramedullary myeloma (EMM) is the presence of soft tissue or viscera in extraosseous locations resulting from hematogenous spread, not contiguous to the involved bone.^23^ (Additional information is provided in the Supplementary Appendix).

### 2.4 Laboratory assessments

The CAR transgene copies in the patient's PBMC were monitored by droplet digital polymerase chain reaction. Evidence of myeloma cell membrane BCMA expression, as determined by MFC or validating immunohistochemistry (IHC) of formalin‐fixed paraffin‐embedded tumor tissue (e.g., bone marrow biopsies or plasmacytoma) with BCMA antibody (Shanghai Yunmai Bio‐Technology Co., AY‐98081R, China). In this study, BCMA + neoplasms were defined as conditions with at least one of the following characteristics: (1) BCMA expression detected in ≥50% of malignant plasma cells by IHC, (2) BCMA expression detected in ≥20% of malignant plasma cells from fresh marrow aspirates by MFC, and (3) mean fluorescence intensity (MFI) of BCMA expression ≥ 1000 as determined by MFC. Serum soluble BCMA and cytokines were performed with enzyme‐linked immunosorbent assays. Cytogenetic and genomic aberrations, including del(17p) and TP53 mutations, were examined by interphase fluorescence in situ hybridization and targeted next‐generation sequencing. Detailed methods were described previously.^24^, ^25^ Schematic diagrams of lentiviral vector and pre‐clinical investigation using cell line and animal models were conducted (Figures S1, S2, and S3 in the Supplementary Appendix).

### 2.5 Statistical analysis

The analysis of categorical variables was performed using Clopper‐Pearson 95% confidence interval (CI) and Fisher's exact test. Wilcoxon rank‐sum test was applied to continuous variables. The probabilities of OS and PFS were estimated utilizing the Kaplan‐Meier method and were compared using the log‐rank test. Cox regression was used to assess the association between factors and survival. All statistical analysis and visualization were performed with the software packages R 3.5.2. *p‐*value < 0.05 was considered statistically significant.

## 3 RESULTS

### 3.1 Patient characteristics

Between February 22, 2017 and June 25, 2018, a total of 28 R/R MM patients and two primary PCL patients were enrolled in this study (Figure 1) and underwent leukapheresis. The manufacturing of anti‐BCMA CAR T cells was successful for 100% of the patients. No patients received bridging therapy during the manufacturing window. The patient characteristics are summarized in Table 1. The median age was 55 years (range = 34–65 years). Note that 43.3% of the patients were females. The median time from diagnosis to infusion was 44.5 months (range = 22–151 months). Subjects had a median of four (range = 3–11) prior lines of therapy, with 100% refractory to a proteasome inhibitor (PI), 83.3% refractory to lenalidomide, and 13.3% refractory to daratumumab.

**TABLE 1:** Baseline characteristics of the patients

**Variables**	**Cohort's value (*n* = 30)**
Median age (range) ‐ year	55 (34–65)
Male sex ‐ number (%)	17 (56.7%)
Median time since diagnosis (range)*	44.5 (22–151)
EMM and sPCL ‐ number (%)	14 (46.7%)
Primary plasma cell leukemia (pPCL) ‐ number (%)	2 (6.7%)
Prior lines of therapy ‐ median (range)	4 (3–11)
Bridging therapy ‐ number (%)^†^	0
Previous auto‐SCT treatment ‐ number (%)	11 (36.7%)
Previous therapies (refractory)‐ number (%)	
Bortezomib	30 (100%)
Lenalidomide	25 (83.3%)
Thalidomide	19 (63.3%)
Carfilzomib	4 (13.3%)
Daratumumab	4 (13.3%)
Ibrutinib	1 (3.3%)
Durie‐Salmon stage^‡^	
IA ‐ number (%)	1 (3.6%)
IIA ‐ number (%)	1 (3.6%)
IIIA ‐ number (%)	26 (92.8%)
ISS stage ^$^	
I ‐ number (%)	10 (35.7%)
II ‐ number (%)	10 (35.7%)
III ‐ number (%)	8 (28.6%)
M protein type ‐ number (%)	
IgA‐kappa	3 (10%)
IgA‐lambda	1 (3.3%)
IgD‐lambda	1 (3.3%)
IgG‐kappa	8 (26.7%)
IgG‐lambda	10 (33.3%)
Light chain‐kappa	3 (10%)
Light chain‐lambda	4 (13.3%)
High risk cytogenetics ‐ number (%)^§^	24 (80%)
del(17p) ‐ number (%)	4 (13.3%)
TP53 mutations ‐ number (%)	2 (6.7%)
BCMA expression rate (median, range)	92.6% (20.1%–98.9%)
BCMA mean FI (median, range)	2092 (303–12516)
Serum free BCMA (median, range) ‐ pg/ml	1928.6 (513.3–2963.4)

Abbreviations: BCMA, B‐cell maturation antigen; EMM, extramedullary myeloma; pPCL, primary plasma cell leukemia; sPCL, secondary plasma cell leukemia.

* The time between the initial diagnosis and screening for the study.

^†^ Bridging therapy was administered after leukapheresis and before lymphodepletion.

^‡^ The staging of 28 relapsed/refractory multiple myeloma at screening.

^§^ High risk was defined by the Mayo stratification of myeloma and risk‐adapted therapy (mSMART) consensus guidelines 2013 at the screening.

A total of 46.7% of the patients had EMM and PCL. A total of 36.7% received prior auto‐SCT treatment. Visualization by box plots and pie charts of patient characteristics are available as Figures S4 and S5 in the Data Supplements. Detailed patient information was provided in Tables S1–S4 in the Supplementary Appendix. Patients received administration of CAR‐BCMA T cells at a median dose of 11.2 × 10^6^ CAR+ cells/kg (range = 5.4–25.0 × 10^6^ CAR+ cells/kg). The percentage of infusion CD3+ cells expressing BCMA CAR was 46.4% (range = 22.6% – 70.4%).

### 3.2 Adverse effects

All 30 patients had AE, and ≥ grade 3 events occurred in all of them. No fatal adverse effects occurred during the trial. A total of 44 different types of AEs were recorded in at least one of the 30 patients, which are summarized in Table 2. Treatment‐related AEs of each patient and the treatment for CRS management are shown in Table S5 in the Data Supplements.

**TABLE 2:** Adverse events (AEs) in 30 patients treated with anti‐BCMA CAR T Cell

**Adverse event**	**Grade 1–2**	**Grade 3**	**Grade 4**	**Any grade**
	**Number of patients (percent)**			
Hematologic				
Leukopenia	1 (3.3)	3 (10)	26 (86.7)	30 (100)
Neutropenia	1 (3.3)	3 (10)	26 (86.7)	30 (100)
Lymphopenia	0	0	30 (100)	30 (100)
Anemia	7 (23.3)	23 (76.7)	0	30 (100.0)
Thrombocytopenia	6 (20.0)	3 (10.0)	21 (70.0)	30 (100.0)
Gastrointestinal				
Belching	4 (13.3)	0	0	4 (13.3)
Bloating	4 (13.3)	0	0	4 (13.3)
Diarrhea	2 (6.7)	0	0	2 (6.7)
Nausea	5 (16.7)	0	0	5 (16.7)
Vomiting	15 (50)	0	0	15 (50)
Respiratory				
Upper respiratory infection	6 (20.0)	0	0	6 (20.0)
Cough	7 (23.3)	0	0	7 (23.3)
Lung infection	3 (10.0)	0	0	3 (10.0)
Cytokine release syndrome	24 (80)	5 (16.7)	0	29 (96.7)
Neurologic toxic effect	1 (3.3)	0	0	1 (3.3)
Other				
Atrial fibrillation	0	1 (3.3)	0	1 (3.3)
Heart failure	0	0	3 (10)	3 (10)
Sinus tachycardia	29 (96.7)	0	0	29 (96.7)
Fatigue	4 (13.3)	0	0	4 (13.3)
Fever	23 (76.7)	6 (20)	0	29 (96.7)
Generalized edema	2 (6.7)	0	0	2 (6.7)
Malaise	11 (36.7)	0	0	11 (36.7)
Activated partial thromboplastin time prolonged	24 (80)	0	0	24 (80)
Alanine aminotransferase increased	2 (6.7)	3 (10)	0	5 (16.7)
Aspartate aminotransferase increased	5 (16.7)	1 (3.3)	0	6 (20)
Blood bicarbonate decreased	19 (63.3)	0	0	19 (63.3)
Blood bilirubin increased	4 (13.3)	0	1 (3.3)	5 (16.7)
Blood lactate dehydrogenase increased	22 (73.3)	0	0	22 (73.3)
Cardiac troponin I increased	2 (6.7)	0	0	2 (6.7)
Cholesterol high	8 (26.7)	0	0	8 (26.7)
Electrocardiogram corrected QT interval (QTc) prolonged	5 (16.7)	1 (3.3)	0	6 (20.0)
Creatinine increased	5 (16.7)	1 (3.3)	0	6 (20.0)
INR increased	19 (63.3)	0	0	19 (63.3)
Hypercalcemia	8 (26.7)	0	0	8 (26.7)
Hyperkalemia	0	0	1 (3.3)	1 (3.3)
Hypernatremia	1 (3.3)			1 (3.3)
Hypokalemia	19 (63.3)	0	0	19 (63.3)
Hyponatremia	19 (63.3)	1 (3.3)	0	20 (66.7)
Confusion	1 (3.3)	0	0	1 (3.3)
Delirium	1 (3.3)	0	0	1 (3.3)
Restlessness	1 (3.3)	0	0	1 (3.3)

Hematologic toxic effects were the most common events of any grade during treatment (neutropenia, lymphopenia, anemia, thrombocytopenia, and leukopenia, all in 100%). The most common ≥ grade 3 AEs were lymphopenia (in 100%, all as grade 4). Among patients who had cytopenia of grade 3 or higher, 79.3% recovered to an absolute neutrophil count of at least 1.0 × 10^9^/L and 54.2% to a platelet count of at least 50 × 10^9^/L within 1 month; however, delayed recovery from cytopenia was observed. The median time from infusion to recovery of an absolute neutrophil count of at least 1.0 × 10^9^/L was 11 days (95% CI, 9–25). Recovery to a platelet count of at least 50 × 10^9^/L occurred in a median of 27.5 days (95% CI, 14 to NA) (Figure S6). At the 3‐month follow‐up, 96.7% recovered to an absolute neutrophil count of at least 1.0 × 10^9^/L and 86.7% to a platelet count of at least 50 × 10^9^/L (Table S6).

Other AE types with ≥grade 3 events included atrial fibrillation (one grade 3 event), heart failure (three grade 4 events), fever (six grade 3 events), increased alanine aminotransferase (three grade 3 events), increased aspartate aminotransferase (one grade 3 event), increased blood bilirubin (one grade 3 events), prolonged QT interval (one grade 3 event), increased creatinine (one grade 3 event), neutropenia (three grade 3 and 26 grade 4 events), thrombocytopenia (three grade 3 and 21 grade 4 events), leukopenia (three grade 3 and 26 grade 4 events), and hyponatremia (one grade 3 event).

The neurologic toxic effect was observed in one patient with grade 1. Three days post‐CAR T cell infusion, patient 12 experienced brief hallucinations and cloudiness of consciousness. After giving dexamethasone 10 mg and mannitol 125 ml, the patient regained consciousness within 30 min. The dexamethasone and mannitol were continued to be used twice in the following 48 h, and the neurologic toxic effects were fully effectively controlled.

CRS occurred in 29 patients, with five of them having grade 3 CRS. Grade 1–2 CRS events were managed by supportive treatment or glucocorticoids. The symptoms of grade 3 CRS events included heart failure (in three patients), atrial fibrillation (in one patient), and neurologic disorders (in one patient). Continuous renal replacement therapy, as well as diuretic medications, was administered to the three patients presenting grade 3 CRS with heart failure and edema. CRS‐associated atrial fibrillation that occurred in one patient was controlled by intravenous amiodarone. Twelve patients (40.0%) received glucocorticoids for CRS‐related toxicities, and one patient (3.3%) received vasopressor. Five patients (16.7%) with abnormally elevated ferritin levels who may progress to hematopoietic syndrome received plasma exchange. After applying glucocorticoids, continuous renal replacement therapy, or plasma exchange, the patients' CRS grades were reduced by one grade within a week. No patient received tocilizumab. We assessed the serum levels of IL‐6 and ferritin before BCMA CAR T cell infusions and multiple time points after infusions. The peak fold‐increase over the before‐treatment level of ferritin but not IL‐6 was associated with grade 2/3 CRS (Figure S7A). The total CAR‐T doses showed no significant effect on the occurrence and severity of CRS (Figure S7B).

### 3.3 Efficacy

**FIGURE 3:**
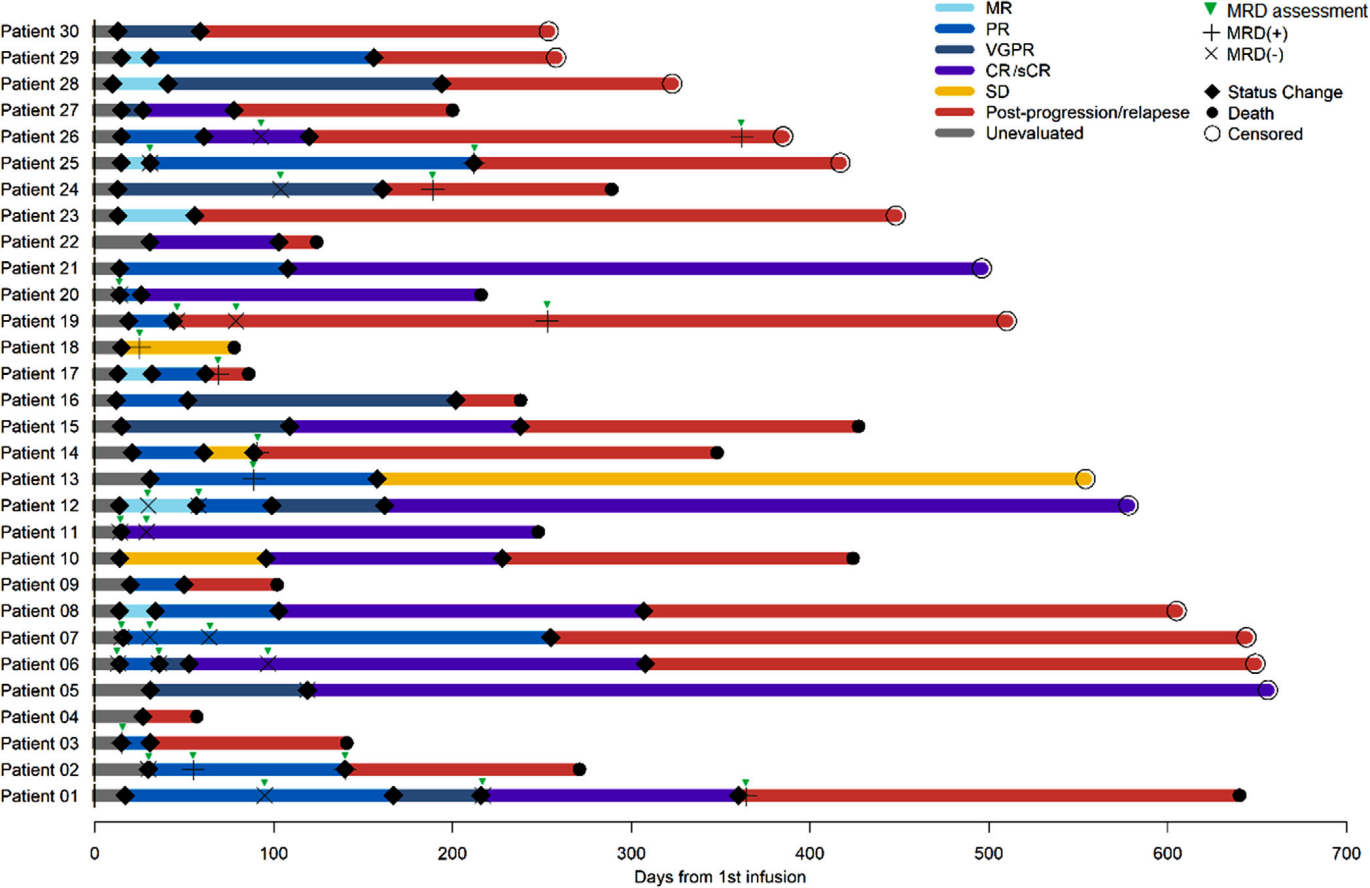
Follow‐up of 30 patients treated with anti‐BCMA CAR T cells. Duration corresponding to different response statuses was represented as the length of colored bars for each patient. Time points of status, change, death, and censoring were also marked Abbreviations: CR, complete response; PD, progressive disease; PR, partial response; sCR, stringent complete response; SD, stable disease; VGPR, very good partial response.

**FIGURE 4:**
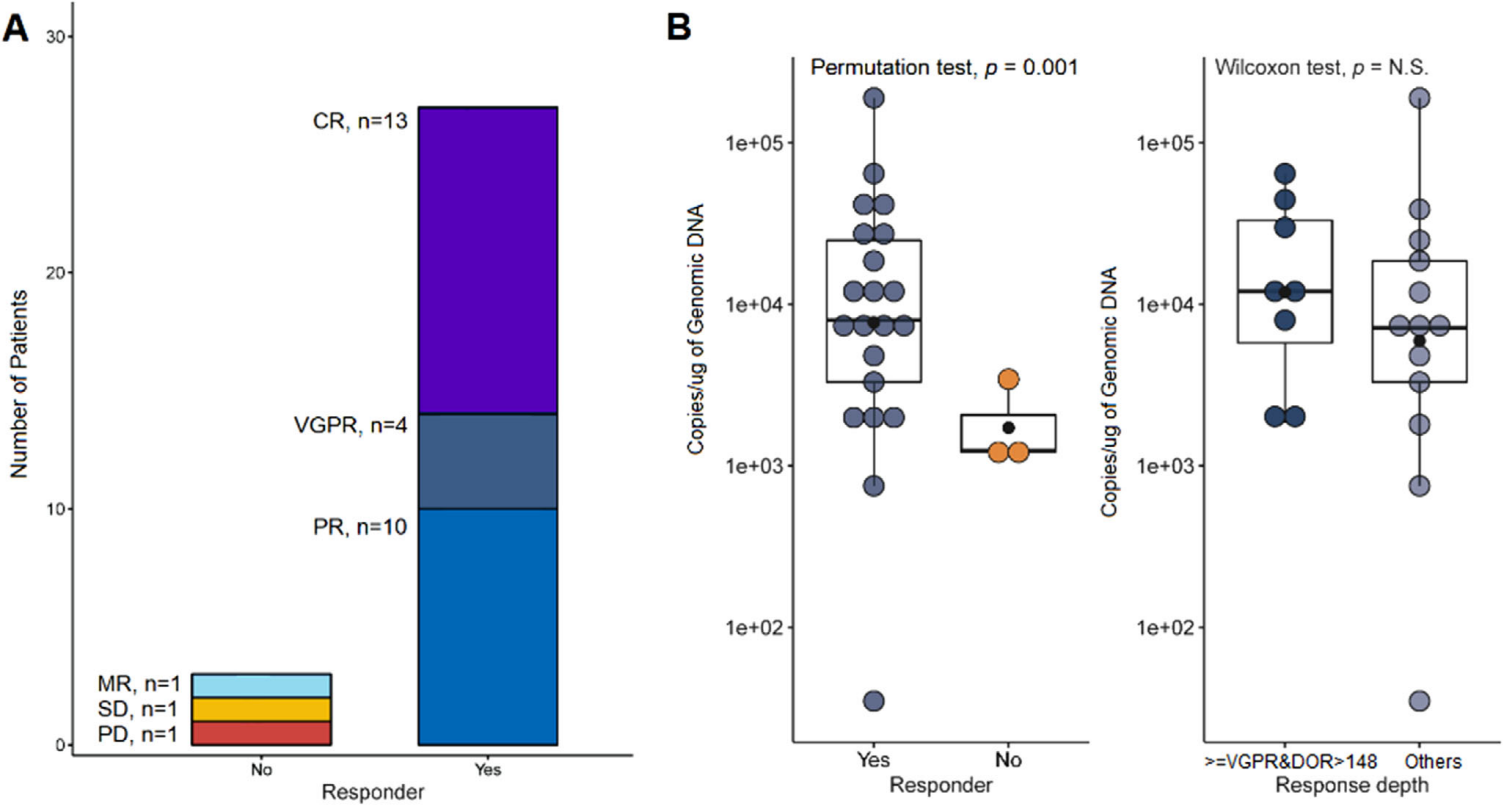
Response profile of 30 patients treated with anti‐BCMA CAR T cells. (A) Response distribution in the 30 patients. Ninety percent of them were responders, including 56.7% showing ≥VGPR. (B) Left: *C*_max_ of transgenic vector copy level between responder and non‐responders; Right: Comparison of *Cmax* between patients with achieved ≥VGPR and DOR > median (148 days) and other responders (≤PR and DOR ≤148 days). Responders had significantly higher *C*_max_ than non‐responders, and there was a trend that deep responders were having higher *C*_max_

**FIGURE 5:**
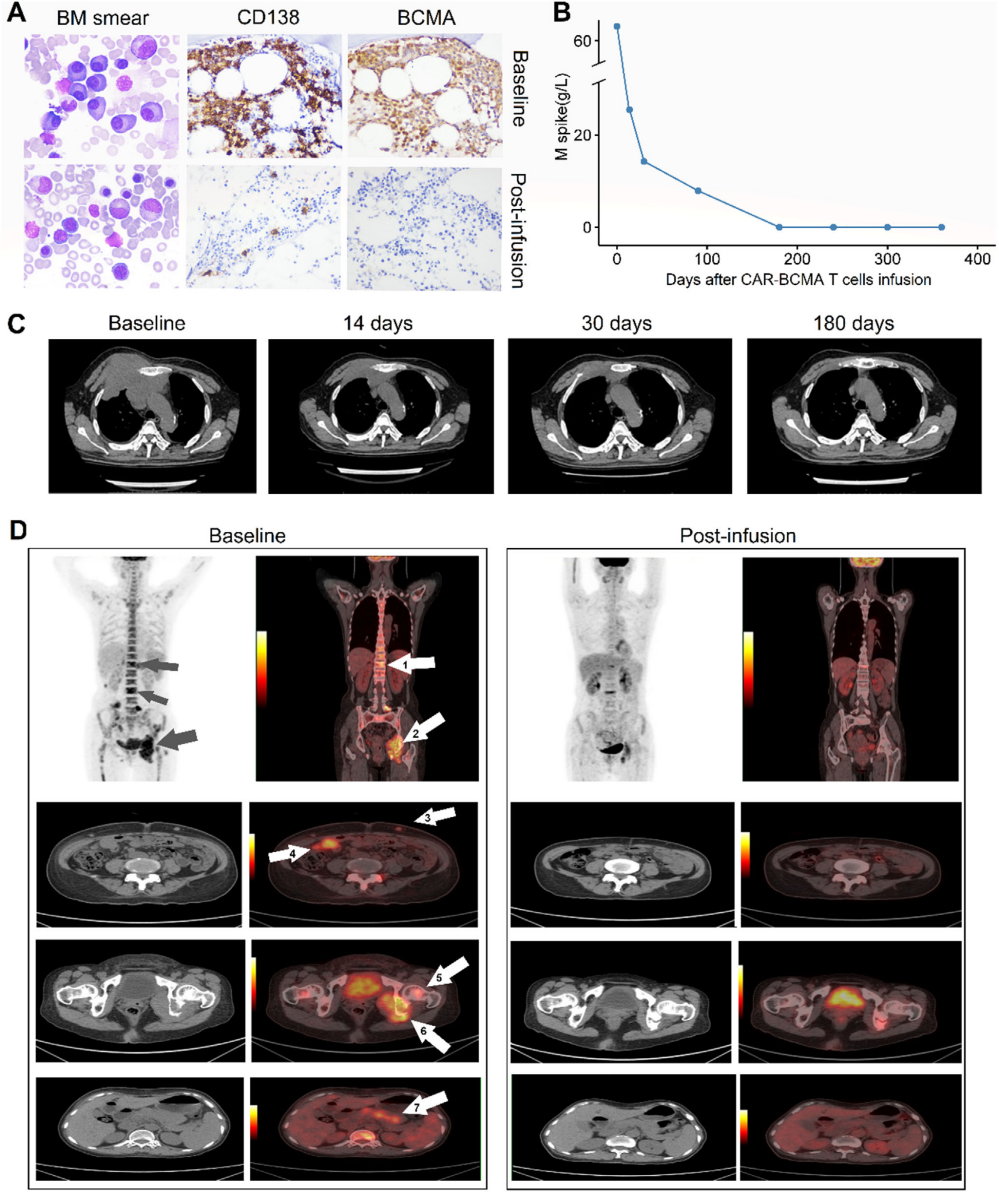
Representative images are showing the anti‐myeloma activity of anti‐BCMA CAR T cells. (A) Patient 1, plasma cells were evaluated in bone marrow smear and core biopsies by CD138 and BCMA IHC staining before CAR‐BCMA treatment (baseline) and 6 months after anti‐BCMA CAR T cells infusion (post‐infusion). (B) Patient 1, longitudinal dynamics of the serum M spike after anti‐BCMA CAR T cell infusion. (C) Patient 16, computerized tomography imaging of the extramedullary disease. The sizeable soft tissue plasmacytoma in the left chest was significantly reduced in size after anti‐BCMA CAR T cell infusion. (D) Patient 28, positron emission tomography imaging of the multiple lesions of this patient with primary plasma cell leukemia and numerous extramedullary involvements (grey arrows, and white arrows 1–7). At day 60 post‐CAR T infusion, the masses on the skin (white arrow 3), abdominal cavity (white arrow 4), next to the left ischial bone (white arrows 5 and 6), and pancreas (white arrow 7) had nearly all disappeared

Patients were assessed for clinical response and followed up to 656 days (median follow‐up, 385 days) by the latest follow‐up review (March 31, 2019). Each patient's response and survival profile were visualized as a bar chart in Figure 3. The objective response rate (ORR) was 90% (95% CI, 84.3%–93.8%; Figure 4A). A response equal to or better than very good partial response (VGPR) was achieved in 56.7% (95% CI, 38.8%–72.9%) of the patients, with 43.3% of the patients having a CR. The ORR was 63.3% at 3 months (Table S7). Representative imaging observations, including one patient with primary PCL and numerous extramedullary involvements, demonstrated the antimyeloma activity of BCMA CAR T cells (Figure 5).

The number of previous treatment lines (>6 vs. ≤6) was the only statistically, significant predictor of ≥VGPR response, with patients with more than six previous treatment lines having less chance of having a ≥VGPR response (Figure S8A). The total CAR T doses showed no significant effect on the best response, PFS, and OS (Figures S8A and S8B). Although there was a trend that higher transduction efficiency in CR patients than others (Wilcoxon test, *p* = 0.08), no statistically significant difference in either transduction efficiency, days of culturing, or cytolysis rates were observed among patients by response status (Kruskal‐Wallis test, *p* = 0.24, 0.83, and 0.17, respectively; Figure S8C). We also found no correlation between these factors and PFS, OS (Figure S8D), and previous treatment lines (Figure S8E).

A total of 17 patients could be evaluated for MRD status in the bone marrow at certain time points (Figure 3). Twelve of 16 who had a response (75%) were MRD‐negative at 10^−4^. Among the 16 patients who achieved partial response (PR) or better, the duration of response (DOR) of MRD‐positive patients was significantly shorter than that of MRD‐negative patients (35 days vs. 217.5 days, *p* = 0.020, Figure S9A).

Among the responders, the DOR was 148 days (range = 16–625 days). The best response achieved (≥VGPR vs. PR) was the only significant predictor of DOR (median DOR, 202 vs. 75 days, *p* = 0.001; Figure S9B). To effectively evaluate CAR T cells' persistence, the CAR transgene copies were monitored in the patient's PBMC by digital droplet PCR. The response was associated with actual CAR T cell exposure strength, with responders showing significantly higher *C*max vector transgene copies than non‐responders (*p* = 0.001; Figure 4B left). There was also a trend of higher *C*max vector transgene copies in patients who achieved ≥VGPR and DOR > median (148 days) compared to other responders (≤PR and DOR ≤148 days) (Figure 4B right). High blood CAR+ cell levels were associated with antimyeloma effectiveness. Responses Peak blood CAR+ cell levels were higher in responders versus nonresponders (*p* = 0.025, Wilcoxon rank‐sum test; *n* = 25 vs. 3) (Figure S10).

**FIGURE 6:**
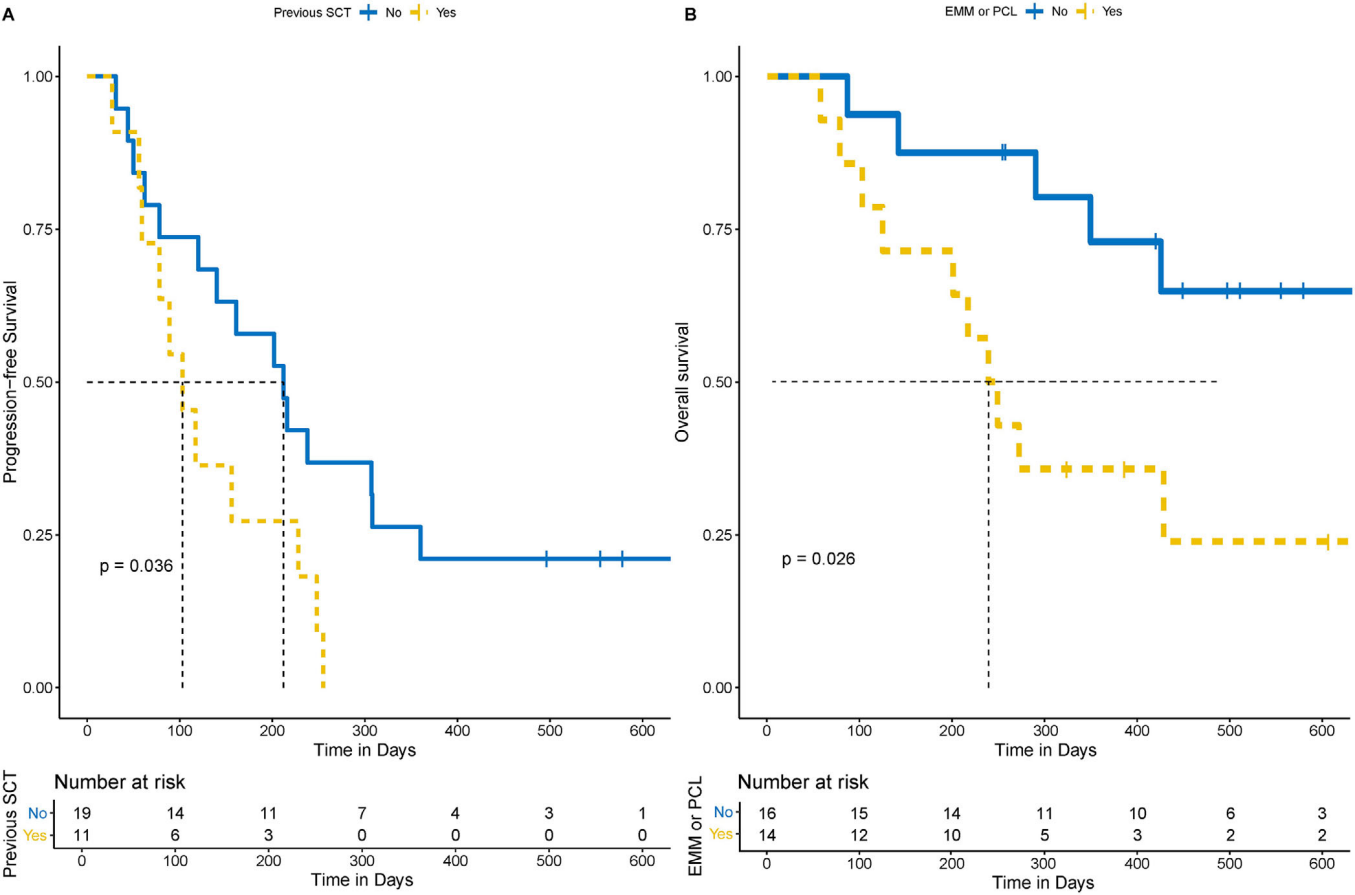
Prognostic factors in 30 patients treated with anti‐BCMA CAR T cells. (A) Patients with previous SCTs showed poorer PFS compared to those without; (B) Patients with EMM/PCL showed worse OS compared to those without

A total of 23 patients (76.7%) had disease progression during the follow‐up, and a total of 16 patients (53.3%) died during the follow‐up. We examined the MFI of BCMA expression in seven MM patients who relapsed or progressed after CAR T treatment. Three patients showed decreased or negative BCMA expression (Table S8). The median PFS of all subjects was 158 days (95% CI, 103–248 days; Figure S11A), and the median OS of all subjects was 427 days (95% CI, 271 to NA days; Figure S11B). According to Cox regression analysis, previous auto‐SCT treatment (yes vs. no) was a significant predictor for PFS (*p* = 0.036, Figure 6A). Although patients presenting EMM/PCL showed significantly worse OS compared to others, the median OS reached 243 days (*p* = 0.026, Figure 6B). All other factors did not have a significant association with PFS or OS (Figure S8B).

The serum BCMA level was measured at baseline and regularly post‐infusion. A significant reduction of serum BCMA level was observed 1‐month post‐infusion compared to baseline (*p* < 0.001; Figures S12A and S12B). Positive and significant correlations between serum BCMA and M/light chain proteins were also observed (M protein, *r* = 0.65, *p* < 0.001; light chain, *r* = 0.73, *p* = 0.016; Figure S12C).

## 4 DISCUSSION

Although these results are preliminary, and the sample size is not very large, the reported activity with anti‐BCMA CAR T‐cells therapy shows potential superiority to other salvage therapies for R/R MM. A multicenter phase 2 study in patients with R/R MM from the Japanese MM‐011 trial showed that patients aged ≤65 years who received pomalidomide plus dexamethasone had an ORR of 47%, and none of the five patients with extramedullary disease achieved a response, with three of them maintaining SD of short duration.^26^ In a randomized Phase III trial, the median PFS with pomalidomide plus low‐dose dexamethasone for R/R MM patients was 4.0 months.^27^ In another phase 2 study, SIRIUS, 106 patients with refractory MM received 16 mg/kg daratumumab, whose RR/CR/VGPR rates were 29.2%/2.8%/9.4%, respectively, and median PFS was 3.7 months.^28^ In an open‐label, single‐arm study, patients treated with isatuximab (another anti‐CD38 antibody) had a median PFS of 3.65 months, and the ORR was 24.3%.^29^ The combination of selinexor and dexamethasone has an ORR of 21% in patients with heavily pretreated refractory myeloma with limited therapeutic options.^30^ In a dose‐escalation and expansion phase 1 trial of a BCMA‐directed antibody (GSK2857916) drug conjugate, 60% of the patients achieved an objective response.^31^

Results from previously published BCMA CAR T trials showed that around 33%–88% of RRMM patients had objective anti‐myeloma responses^12^, ^15^, ^32^, ^33^, ^34^ and a median PFS of approximately 7–15 months^12^, ^15^, ^33^, ^34^ after treatment with anti‐BCMA CAR T cells. In our study of anti‐BCMA CAR T cells involving patients with heavily pretreated RRMM and primary PCL, 27 of the 30 patients obtained responses of partial response or better. The ORR was 90%, with 56.7% ≥ VGPR and 43.3% CR. Interestingly, there was a significant difference between patients with >6 lines of therapy versus <6, suggesting an earlier CAR T cell treatment may bring additional benefits to the patients.

The results were having to do with the depth of response (PR vs. VGPR), and DOR in MRD negative versus positive patients, and higher CAR expression in responders are consistent with other studies. High response rates (including the CR/sCR, VGPR, and PR) were also observed in patients with a baseline high‐risk cytogenetic profile (23/24, 95.8%), or EMM and PCL (13/14, 92.8%) in our study. There are five patients with PR who have sustained remission for a short period and may be likely due to the fludarabine and cyclophosphamide lymphodepleting chemotherapy's contribution. Our comprehensive data analyses showed that the number of previous treatment lines, previous stem cell transplantation, and presence of EMM or PCL was the only significant factor associated with the best response, PFS, and OS, respectively. These results need confirmation in future studies.

Factors that can preclude durable remissions following CAR T cell therapy include CAR T cell manufacturing issues, limited CAR T cell expansion and/or persistence, various resistance mechanisms, and toxicities.^35^ Several putative mechanisms, including antigen escape, T cell intrinsic mechanisms, tumor micro environment‐mediated suppression, and host anti‐car immunity, may contribute to the lack of persistence of CAR T cells in vivo.^35^ The results of this study showed that the transduction efficiency, days of culturing, or cytolysis rates were not related to the patient's treatment response, PFS, OS, or prior treatment lines. The median PFS of all subjects was only 5.2 months days and may be related to the one or several factors and mechanisms mentioned above. This study enrolled more EMM or PCL. The highly heterogeneous and continue evolving extramedullary lesions might contain or give rise to clones capable of escaping anti‐BCMA CAR T treatment.^23^ Besides, the micro‐environment of extramedullary lesions is relatively less accessible for CAR T cells to penetrate and persist.^36^

This study also included two patients with primary PCL, which to our knowledge, was the first report. Anti‐BCMA CAR T cell therapy also exerted a therapeutic effect for primary PCL. One had CR with a PFS of 307 days. The other had VGPR with a PFS of 117 days. Due to this trial's limited sample size, this phenomenon should be further validated in a study with more patients.

At present, the treatment of R/R primary PCL is a big challenge. Moreover, most of the current clinical researches, including CAR T studies, exclude PCL, and so it is underrepresented and therefore is an unmet need. Additionally, whether treatment efficacy could be further improved if this approach is combined with existing treatment strategies (e.g., lenalidomide or immune checkpoint inhibitors or auto‐hematopoietic stem cell transplantation (HSCT)) remains unknown. We also suggest that anti‐BCMA CAR T cell treatment can eventually be combined with other effective therapeutics to treat patients with EMM and PCL for achieving a better result in future trials.

The level of plasma BCMA decreased rapidly within the first‐month post‐infusion and was positively associated with whole immunoglobulin M protein/tumor‐associated serum‐free light chains in our study. Serum BCMA levels remained stable or slightly increased in patients with stable disease or progressive disease after anti‐BCMA CAR T cells infusion. Twelve of the 17 patients evaluable for MRD had MRD‐negative status (≤10^−4^ nucleated cells by flow). The DOR time of four MRD‐positive patients who achieved PR was significantly shorter than that of 12 MRD‐negative patients who achieved PR or better. Similar to most research results,^12^ higher *C*max vector transgene copies and peak levels of CAR T cells were associated with clinical anti‐malignancy responses in our current work. Higher *C*max vector transgene copies and peak levels of CAR T cells were observed in responders (≥PR) versus non‐responders. Furthermore, a trend of higher *C*max vector transgene copies was observed in patients with deep response compared to others, although statistical significance was not achieved.

CRS in our study was mostly of grade 1 or 2 (80%); the five grade 3 events (in 16.7% of the patients) resolved within 48 h. The overall frequency of grade 1 neurologic toxic effects was also low (3.3%) resolved within 48 h after treatment. As a common AE of CAR T cell therapy, the neurologic toxic effect was found in 1.8%–42% of previous anti‐BCMA CAR T trials.^12^, ^15^, ^34^ Compared with the other BCMA CAR T study, which was reported previously using CD28 as a costimulatory molecule,^15^ the toxicities of our anti‐BCMA CAR T cells are indeed lower. Still, the mechanism is unclear and needs further study. Hematological toxicities were the most common and most severe and may be related to lymphodepleting chemotherapy and anti‐BCMA CAR T cell infusions. The dose of FC was higher than that employed in other anti‐BCMA CAR T trials,^12^, ^14^, ^15^, ^33^, ^34^ and a reduced dose of lymphodepletion regimens for the next phase II trials is under consideration. Our study findings indicate a favorable safety profile of anti‐BCMA CAR T cells in malignant plasma cell disease patients.

Although all patients in the current study received at least three lines of prior therapies, and most patients were refractory to both bortezomib and lenalidomide, the cohort appears to be less heavily pre‐treated compared to other BCMA CAR T trials,^12^, ^37^ due to limited availability of certain drugs in China, such as carfilzomib, pomalidomide, and daratumumab. The proportion of patients who previously received auto‐HSCT was also relatively low but comparable to the average level in Chinese MM patients.^38^, ^39^ Some MM patients who had relapse/progressive disease had decreased levels of the MFI of BCMA expression, suggesting a probable self‐adaption and escape mechanism. BCMA‐negative or BCMA‐low MM cells are implicated as a reservoir of treatment‐resistant disease preceding relapse in recent clinical investigations of cellular therapies and maybe one of several mechanisms responsible for relapse.^14^, ^15^ An approach to mitigate BCMA escapes‐mediated relapse is through simultaneous targeting of an additional antigen such as G protein‐coupled receptor class C group 5 member D (GPRC5D).^40^ Therefore, on the one hand, anti‐plasma cell CAR T therapies should be considered to combinational antigen targeting of multiple tumor‐associated antigens in future clinical practice. On the other hand, the tumor cells at relapse or progression still expressed BCMA, suggesting that enhancing the efficacy and decreasing immunogenicity of CARs should be considered as a starting point for the novel CAR design.

In conclusion, anti‐BCMA CAR T cells showed promising safety and efficacy in R/R MM. R/R primary PCL patients may benefit from CAR T cell treatment, although it is true that the duration of response is short.

## CONSENT FOR PUBLICATION

The authors have obtained consent to publish from the participant to report patient data.

## CONFLICT OF INTEREST

The authors declare that there is no conflict of interest.

## AUTHOR CONTRIBUTIONS

Jianfeng Zhou designed and supervised the clinical study. Chunrui Li and Jian Li analyzed data, wrote, and revised the manuscript. Chaojiang Gu, Shangkun Zhang, and Tongcun Zhang supervised the CAR T cell production. Hao Xu, Wenyue Cao, and Na Wang conducted preclinical validation and quality control. Qiuxiang Wang and Lijun Jiang collected clinical data. Jue Wang and Di Wang performed statistical analyses. Yi Xiao, Jinhuan Xu, Xiaoxi Zhou, Zhenya Hong, Liang Huang, Li Meng, and Yang Cao enrolled patients and took care of the patients. Liting Chen, Xia Mao, Min Xiao, and Wei Zhang contributed to laboratory tests and response monitoring of the patients. Yimei Que analyzed data, wrote, and revised the manuscript.

## AVAILABILITY OF DATA AND MATERIALS

The datasets supporting the conclusions of this article are included within the article and additional files.

## Supporting information

Appendix

Protocol
