# Supplementary material for: A phase I study of anti‐BCMA CAR T cell therapy in relapsed/refractory multiple myeloma and plasma cell leukemia
Source: Clin Transl Med. 2021 Mar 9;11(3):e346. doi: 10.1002/ctm2.346 (PMC7943908; doi:10.1002/ctm2.346)
Supplement: Supplementary file 1 — Appendix [file CTM2-11-e346-s002.docx]

**Supplementary Appendix**

This appendix is provided by the authors for additional information about their work.

Supplements to Chunrui Li*, et al.* **A phase I study of anti-BCMA CAR T Cell Therapy in relapsed or refractory multiple myeloma and plasma cell leukemia**

**SUPPLEMENTAL MATERIALS**

**CONTENTS:**

**Supplementary methods**

Quality-control assays

**Supplementary tables**

Table S1. Baseline Characteristics of the Patients, Cells Dose and Percentage of infused T cells that expressed CAR-BCMA

Table S2. Therapies received before CAR-BCMA T-cell protocol enrollment

Table S3. Targeted next-generation sequencing and Sanger sequencing

Table S4. Interphase fluorescence ***in situ*** hybridization (iFISH)

Table S5. Adverse Events and CRS for patients receiving CAR-BCMA T cells

Table S6. Adverse Events of the hematological toxicities for patients receiving anti-BCMA CAR T cells

therapy at three months

Table S7. The response status for patients receiving anti-BCMA CAR T cells therapy at three months

Table S8. Comparison of BCMA median fluorescence intensity between baseline and relapse

**Supplementary figures**

Figure S1. Chromosomal distribution of LV integrants in anti-BCMA CAR-T cells

Figure S2. CAR-BCMA T cells exhibited antileukemic effects in vitro

Figure S3. BCMA T-cell eradiated U266 cells in a xenograft mouse model

Figure S4. Boxplots with binned dots showing the distribution of investigated continuous variables

Figure S5. Pie charts showing the distribution of categorical variables investigated

Figure S6. Time to Recovery of Grade 3/4 Cytopenias

Figure S7. Peak fold-increase over baseline of ferritin and IL-6 levels by CRS

Figure S8. Forest plots showing an association between factors and efficacy outcomes

Figure S9. The DOR of patients

Figure S10. Peak blood CAR+ cell levels were higher in responders versus nonresponders

Figure S11. Long-term outcome of 30 patients

Figure S12. Serum BCMA level dynamics and its correlation with M proteins

**Supplemental references**

**SUPPLEMENTAL METHODS**

**Quality-control assays**

T cells were stained with Alexa Fluor 647 labeled protein L or rabbit anti-mouse-F (ab) 2 antibodies (Jackson ImmunoResearch) for assessing the transduction efficiency. The apoptosis assay was conducted using an Annexin V Apoptosis Detection Kit (BD Biosciences) according to the manufacturer's instructions. Data acquisition was conducted on a cytometer. The calcein release assay was performed for determining the tumor-killing efficiency of CAR T-cell as previously reported ^1,2^. CAR gene-specific minor groove binder (MGB) probe was designed by Primer Express 3.0. Absolute quantification of the CAR gene in peripheral blood was determined by droplet digital polymerase chain reaction (ddPCR). Genomic DNA was isolated from whole blood samples obtained at serial time points before and after the CAR T-cell infusion.

**Supplemental Table 1.** **Baseline characteristics of the patients, cells dose, and percentage of infused T cells that expressed CAR-BCMA**

| **Patient No.** | **Gender** | Age (Years) | Malignancy | Monoclonal Protein | **Durie-Salmon stage** | **ISS stage** | **Prior Lines of Therapy^‡^** | **CAR-T cells/kg**  **(**$\boldsymbol{\times}$**10^6^)** | **Transduction**  **efficiency ^&&^** | **Days of culture** | **Cytolysis rates (%),**  **E: T = 25:1** |
| --- | --- | --- | --- | --- | --- | --- | --- | --- | --- | --- | --- |
| 1 | F | 44 | MM | IgG-κ | IIIA | I | 8 | 11.2 | 63.3% | 14 | 90.3% |
| 2 | F | 55 | sPCL | IgG-κ | IIIA | II | 4 | 10.0 | 52.9% | 14 | 75.3% |
| 3 | F | 64 | MM | κ light chain | IIIA | III | 3 | 10.0 | 48.5% | 17 | 84.2% |
| 4 | F | 55 | EMM | IgG-λ | IIIA | II | 9 | 11.5 | 34.6% | 12 | 69.9% |
| 5 | M | 48 | MM | IgG-λ | IIIA | I | 5 | 10.5 | 39.0% | 12 | 43.3% |
| 6 | F | 55 | MM | IgG-λ | IIIA | III | 3 | 10.0 | 66.5% | 14 | 84.5% |
| 7 | F | 56 | sPCL | IgG-λ | IIIA | II | 4 | 10.2 | 54.9% | 19 | 91.3% |
| 8 | M | 54 | pPCL | IgG-κ | NE | NE | 3 | 9.5 | 40.8% | 14 | 35.6% |
| 9 | F | 54 | sPCL | λ light chain | IIIA | III | 7 | 9.7 | 41.5% | 14 | 61.8% |
| 10 | F | 59 | MM | IgA-λ | IIIA | III | 4 | 5.6 | 70.4% | 15 | 98.6% |
| 11 | F | 60 | EMM; sPCL | IgA-κ | IIIA | I | 3 | 14.5 | 35.6% | 15 | 100.0% |
| 12 | F | 65 | MM | IgG-λ | IIA | I | 3 | 24.7 | 49.8% | 16 | 54.7% |
| 13 | F | 64 | MM | IgG-κ | IIIA | II | 7 | 21.0 | 45.6% | 21 | 48.1% |
| 14 | M | 57 | MM | IgG-κ | IIIA | III | 7 | 12.3 | 22.6% | 15 | 59.6% |
| 15 | M | 65 | EMM | IgG-κ | IIIA | I | 4 | 19.5 | 41.8% | 16 | 11.5% |
| 16 | M | 62 | EMM | λ light chain | IIIA | II | 4 | 16.5 | 36.5% | 18 | 53.7% |
| 17 | M | 53 | MM | IgG-λ | IIIA | II | 4 | 14.5 | 34.6% | 15 | 7.5% |
| 18 | M | 55 | sPCL | IgG-λ | IIIA | III | 11 | 25.0 | 46.1% | 19 | 63.0% |
| 19 | M | 42 | MM | κ light chain | IIIA | I | 3 | 20.0 | 31.9% | 25 | 12.8% |
| 20 | M | 65 | EMM | IgD-λ | IIIA | III | 5 | 15.0 | 46.6% | 18 | 80.4% |
| 21 | M | 65 | MM | IgA-κ | IIIA | I | 4 | 8.2 | 52.6% | 17 | 30.3% |
| 22 | M | 53 | EMM | λ light chain | IIIA | II | 4 | 5.4 | 57.2% | 15 | 22.8% |
| 23 | M | 34 | MM | IgG-κ | IIIA | II | 3 | 7.5 | 58.2% | 18 | 41.3% |
| 24 | M | 55 | MM | IgG-κ | IIIA | III | 3 | 12.5 | 25.1% | 16 | 12.8% |
| 25 | M | 52 | MM | IgG-λ | IIIA | II | 8 | 20.5 | 44.8% | 14 | NE |
| 26 | M | 34 | EMM | IgA-κ | IA | I | 7 | 17.7 | 39.9% | 15 | NE |
| 27 | M | 61 | EMM | λ light chain | IIIA | I | 6 | 10.5 | 55.6% | 19 | NE |
| 28 | F | 56 | pPCL; EMM | IgG-λ | NE | NE | 3 | 6.1 | 46.7% | 23 | NE |
| 29 | M | 53 | MM | IgG-λ | IIIA | II | 5 | 6.1 | 39.0% | 19 | NE |
| 30 | F | 40 | MM | κ light chain | IIIA | I | 3 | 11.2 | 55.7% | 16 | NE |

^‡^: The number of prior lines of therapy was calculated by adding all discrete lines of therapy received by each patient. An induction regimen followed by autologous stem-cell transplantation, which includes induction chemotherapy, mobilization chemotherapy, conditioning regimen, and any maintenance therapy, was considered one line of therapy. Radiation was considered a line of therapy. Abbreviations: EMM, extramedullary myeloma; sPCL, secondary plasma cell leukemia; pPCL, primary plasma cell leukemia; ISS, International staging system; NE, not evaluated; Ig, immunoglobulin; F, female; M, male;^&&^Percentage of infusion CD3^+^ cells expressing CAR-BCM: CAR-BCMA was detected on the surface of CD3^+^ T cells by staining with anti-mouse IgG. E:T ratio, Effector to target cell ratio.

**Supplemental Table 2. Therapies received before anti-BCMA CAR T Cells protocol enrollment**

| Patient No. | Treatment | Lines |
| --- | --- | --- |
| 1 | VD→TD→CyBorD→RD→VRD→VAD→CPT Clinical Trial in China→VTD | 8 |
| 2 | VD→VTD→VRD→RD | 4 |
| 3 | VAD→RD→VRD | 3 |
| 4 | VD then HDT+Auto-SCT and TD→VAD→MP→VRD→VD→RVAD→DT-PACE→DCEP→CyBorD | 9 |
| 5 | VD→CyBorD→VAD→TD→VRD | 5 |
| 6 | RVD→CD→ RD | 3 |
| 7 | VD then HDT+Auto-SCT and TD→BIRD →VRD then HDT+Auto-SCT and RD→CyBorD | 4 |
| 8 | VRD→RAD→RD | 3 |
| 9 | VTD→VAD→TD and Chinese herbal medicine→VRD→BIRD→CTD →VCD | 7 |
| 10 | CyBorD then HDT+Auto-SCT and TD→BIRD→VRD→DRD | 4 |
| 11 | CyBorD then HDT+Auto-SCT and RD→ VAD→VRD | 3 |
| 12 | VD→RD→CyBorD | 3 |
| 13 | VD→VMP→Etoposide and Ifosfamide→ MPT →RD→RCD→CyBorD | 7 |
| 14 | VAD then HDT+Auto-SCT and TD→CyBorD→VCD→VMP then HDT+Auto-SCT and RD→DT-PACE→ Bortezomib + Ibrutinib→VRD | 7 |
| 15 | CyBorD→RD→VRD→RTX | 4 |
| 16 | VD→RD→VRD→DRD | 4 |
| 17 | VD→VAD→RD→CyBorD | 4 |
| 18 | PCD→VD→VTD→VRD→PAD→PACE→DAC→TD→MPT→VDD→CyBorD | 11 |
| 19 | VD→VTD→VRD | 3 |
| 20 | VAD→CyBorD →VCD→RD→Etoposide + Ifosfamide | 5 |
| 21 | VTD→VCD→ CyBorD → RD | 4 |
| 22 | VD then HDT+Auto-SCT and RD→VRD→TD→ DRD | 4 |
| 23 | CyBorD→DT-PACE then HDT+Auto-SCT and RD→BIRD | 3 |
| 24 | VD→CyBorD→VTD | 3 |
| 25 | VAD→TD→ CyBorD→RD→VD→ VCD→VRD→CD | 8 |
| 26 | MP→VAD then HDT+Auto-SCT and RD→GNP→RTX→VTD→ EPOCH→Gemox | 7 |
| 27 | VCD then HDT+Auto-SCT and TD→VD→CCD→RD→CRD→DRD | 6 |
| 28 | VAD then HDT+Auto-SCT and RD→RTX→VRD | 3 |
| 29 | DT-PACE then HDT+Auto-SCT and RD→VD→VTD→VRD→CD | 5 |
| 30 | VCD then HDT+Auto-SCT and RD→CTD→CD | 3 |

**Abbreviations**

BIRD: Clarithromycin, Aspirin, Lenalidomide, Dexamethasone

CyBorD: Cyclophosphamide, Bortezomib, Dexamethasone

CCD: Carfilzomib, Cyclophosphamide, Dexamethasone

CD: Carfilzomib, Dexamethasone

CRD: Carfilzomib, Lenalidomide, Dexamethasone

CTD: Thalidomide, Cyclophosphamide, Dexamethasone

DAC: Dexamethasone, Doxorubicin, Cyclophosphamide

DCEP: Dexamethasone, Cyclophosphamide, Etoposide, Cisplatin

DRD: Daratumumab, Lenalidomide, Dexamethasone

DT-PACE: Dexamethasone, Thalidomide, Cisplatin, Doxorubicin, Cyclophosphamide, Etoposide

EPOCH: Etoposide, Prednisone, Vincristine, Cyclophosphamide, Doxorubicin

Gemox: Gemcitabine + Oxaliplatin

GNP: Gemcitabine, Nedaplatin, Prednisone

HDT+Auto-SCT: High dose therapy and autologous stem cell transplantation

MP: Marfan, Prednisone

MPT: Marfan, Prednisone, Thalidomide

PACE: Cisplatin, Doxorubicin, Cyclophosphamide, Etoposide

PAD: Cisplatin, Doxorubicin, Dexamethasone

PCD: Cisplatin, Cyclophosphamide, Dexamethasone

RAD: Lenalidomide, Doxorubicin, Dexamethasone

RCD: Lenalidomide, Cyclophosphamide, Dexamethasone

RD: Lenalidomide, Dexamethasone

RTX: Radiation Therapy

RVAD: Lenalidomide, Bortezomib, Doxorubicin, Dexamethasone

TD: Thalidomide, Dexamethasone

VAD: Bortezomib, Doxorubicin, Dexamethasone

VCD: Bortezomib, Cyclophosphamide, Liposomal doxorubicin

VD: Bortezomib, Dexamethasone

VDD: Bortezomib, Liposomal doxorubicin, Dexamethasone

VMP: Marfan, Prednisone, Bortezomib

VRD: Lenalidomide, Bortezomib, Dexamethasone

VTD: Thalidomide Bortezomib, Dexamethasone

**Supplemental Table 3: Targeted next-generation sequencing and Sanger sequencing**

| Patient No. | Source of sampling | Mutations | Mutant allele fraction | Significance |
| --- | --- | --- | --- | --- |
| 1 | BM | Negative |  |  |
| 2 | BM | Negative |  |  |
| 4 | BM | Negative |  |  |
| 5 | BM | PTEN, c.802G>T, p.Asn268Tyr (p.D268Y) | 11.36 | Uncertain |
| 6 | BM | Negative |  |  |
| 8 | BM | Negative |  |  |
| 9 | BM | BRAF, c.64G>A, p.Asp22Asn (p.D22N) | 36.36 | Uncertain |
| 10 | BM | DIS3, c.2257T>A, p.Tyr753Asn (p.Y753N) | 57.21 | Uncertain |
| 11 | BM | Negative |  |  |
| 13 | BM | Negative |  |  |
| 14 | BM | BRAF, c.1799T>A, p.Val600Glu (p.V600E) | 56.5 | Pathogenic |
|  | BM | DIS3, c.1978A>G, p.Asn660Asp (p.N660D) | 57.2 | Uncertain |
| 15 | Brain | Negative |  |  |
|  | BM | Negative |  |  |
| 16 | Chest | KRAS, c. 35_38delinsCCAC,  p.Gly12_Gly13delinsAlaThr (p.G12_G13delinsAT) | 46.5 | Likely  Pathogenic |
|  | Chest | MAX, c.146C>G, p.Ser49* (p.S49*) | 83.2 | Uncertain |
| 17 | BM | FGFR3, c.818T>G, p.Phe273Cys (p.F273C) | 30.5 | Uncertain |
| 18 | BM | Negative |  |  |
| 19 | BM | BRAF, c.1447A > G, p.Lys483Glu (p.K483E) | 11.4 | Likely  Pathogenic |
| 20 | BM | NRAS,c.35G > T, p.Gly12Val (p.G12V) | 10.0 | Pathogenic |
|  | BM | ACTG1, c.14T > C, p.Ile5Thr (p.I5T) | 11.4 | Uncertain |
|  | Leg | NRAS, c.35G > T, p.Gly12Val (p.G12V) | 85.4 | Pathogenic |
|  | Leg | ACTG1, c.14T > C, p.Ile5Thr (p.I5T) | 90.1 | Uncertain |
| 21 | BM | FGFR3, c.833A > G, p.Tyr278Cys (p.Y278C) | 38.5 | Uncertain |
| 22 | BM | Negative |  |  |
| 24 | BM | KRAS, c. 38G > A, p. Gly13Asp (p. G13D) | 9.7 | Pathogenic |
| 25 | BM | BRAF, c. 1799T > A, p. Val600Glu (p. V600E) | 9.7 | Pathogenic |
| 26 | Lymph node | BRAF, c. 1799T > A, p. Val600Glu (p. V600E) | 41.6 | Pathogenic |
| 27 | BM | HIST1H1E, c.53C > T, p.Thr18Ile (p.T18I) | 66.8 | Uncertain |
| 28 | Skin | KRAS, c.34G > C, p.Gly12Arg (p.G12R) | 3.1 | Pathogenic |
|  | Skin | TP53, c.747G > T, p.Arg249Ser (p.R249S) | 7.8 | Likely  Pathogenic |
|  | BM | KRAS, c.183A > C, p.Gln61His (p.Q61H) | 78.1 | Pathogenic |
|  | BM | TRAF3, c.325G > T, p.Glu109* (p.E109) | 44.6 | Uncertain |
|  | ctDNA | FGFR3, c.2162G > C, p.Cys721Ser (p.C721S) | 11.7 | Uncertain |
|  | ctDNA | KRAS, c.34G > C, p.Gly12Arg (p.G12R) | 2.6 | Pathogenic |
|  | ctDNA | TP53, c.747G > T, p.Arg249Ser (p.R249S) | 8.4 | Likely  Pathogenic |
|  | ctDNA | CCND1, c. 574+2T > A | 9.1 | Uncertain |
| 29 | BM | DIS3, c.2335A > T, p.Ile779Phe (p.I779F) | 43.4 | Uncertain |
| 30 | BM | TRAF3 c. 297+2T > G | 66.8 | Uncertain |
|  | BM | MAX c. 94G > A, p. Glu32Lys (p. E32K) | 67.4 | Uncertain |

BM: Bone marrow; PB: Peripheral blood. ctDNA: circulating cell-free tumor-derived DNA

Next-generation sequencing was not performed for patients 3, 7, 12, and 23.

**Supplemental Table 4:** Interphase fluorescence ***in situ*** hybridization (iFISH)

| Patient No. | Sample source | Genetic abnormalities | Percentage of abnormal cells | Genetic abnormalities | Percentage of abnormal cells | Number of High-Risk Genetic Abnormalities |
| --- | --- | --- | --- | --- | --- | --- |
| 1 | BM | 1q gain | 25 | t (4; 14) | 45 | 2 |
| 2 | BM | 1q gain |  | t (4; 14) |  | 2 |
| 3 | BM | 1q gain | 70 | t (4; 14) | 76 | 2 |
| 4 | BM | 1q gain | 85 | t (4; 14) | 85 | 2 |
| 5 | BM | Tetraploid karyotype |  | 1q gain / t (4; 14) | 60 / 47 | 2 |
| 6 | BM | 1q gain | 80 | t (4; 14) | 80 | 2 |
| 7 | BM | Tetraploid karyotype |  | 1q gain | 80 | 1 |
| 9 | BM | 1q gain | 82 | t (11;14) | 81 | 1 |
| 10 | BM | Tetraploid karyotype |  | 1q gain / t (4;14) | 38  31 | 2 |
| 12 | BM | Del (17p) | 48 | t (11;14) | 30 | 1 |
| 13 | BM | t (11;14) | 81 |  |  | 0 |
| 14 | BM | 1q gain | 15 | Del (17p) | 45 | 2 |
| 15 | BM | Negative |  |  |  | 0 |
| 16 | Chest | 1q gain | 40 |  |  | 1 |
| 17 | BM | 1q gain |  | t (4;14) |  | 2 |
| 18 | BM | IGH rearrangement | 76 |  |  | 0 |
| 19 | BM | 1q gain | 65 | t (11;14) / Del (17p) | 58 /60 | 2 |
| 20 | BM | 1q gain | 80 | IGH rearrangement | 76 | 1 |
| 21 | BM | 1q gain | 90 | t (4;14) | 90 | 2 |
| 23 | BM | 1q gain | 85 | IGH rearrangement | 99 | 1 |
| 24 | BM | 1q gain |  | Del (17p) |  | 2 |
| 25 | BM | 1q gain |  |  |  | 1 |
| 26 | BM | 1q gain | 75 |  |  | 1 |
| 27 | BM | 1q gain |  | IGH rearrangement |  | 1 |
| 28 | BM | 1q gain | 12 | t (4;14) | 12 | 2 |
| 29 | BM | 1q gain | 80 | t (4;14) | 82 | 2 |
| 30 | BM | 1q gain | 60 |  |  | 1 |

BM: Bone marrow; Interphase fluorescence in situ hybridization was not performed for patients 8, 11, and 22.

Supplemental Table 5: Adverse Events and CRS for patients receiving **anti-BCMA CAR T cells therapy**

| Patient | Grade3 | Grade4 | CRS | Treatment for CRS management |
| --- | --- | --- | --- | --- |
| 1 | Anemia  Febrile neutropenia  Alanine aminotransferase increased | Lymphopenia  Neutropenia  Thrombocytopenia  Leukopenia | 1 |  |
| 2 | Anemia  Febrile neutropenia | Lymphopenia  Neutropenia  Thrombocytopenia  Leukopenia | 1 |  |
| 3 | Anemia  Febrile neutropenia | Lymphopenia  Neutropenia  Thrombocytopenia  Leukopenia | 1 | Glucocorticoid |
| 4 | Anemia  Febrile neutropenia  Neutropenia  Thrombocytopenia | Lymphopenia  Leukopenia | 1 |  |
| 5 | Febrile neutropenia  Neutropenia  Leukopenia | Lymphopenia | 1 |  |
| 6 | Anemia  Febrile neutropenia  Fever  Thrombocytopenia | Lymphopenia  Neutropenia  Leukopenia  Hyperkalemia | 2 |  |
| 7 | Anemia  Febrile neutropenia  Lung infection  Electrocardiogram QT corrected interval prolonged | Lymphopenia  Neutropenia  Thrombocytopenia  Leukopenia | 1 |  |
| 8 | Leukopenia | Lymphopenia | 0 |  |
| 9 | Anemia  Febrile neutropenia | Lymphopenia  Neutropenia  Thrombocytopenia  Leukopenia | 1 |  |
| 10 | Anemia  Febrile neutropenia | Lymphopenia  Neutropenia  Thrombocytopenia  Leukopenia | 1 | Glucocorticoid |
| 11 | Anemia  Febrile neutropenia  Alanine aminotransferase increased | Lymphopenia  Neutropenia  Thrombocytopenia  Leukopenia | 1 |  |
| 12 | Anemia  Febrile neutropenia  Fever  Hyponatremia | Lymphopenia  Neutropenia  Thrombocytopenia  Leukopenia | 3 | Glucocorticoid |
| 13 | Anemia  Febrile neutropenia  Fever  Lung infection  Alanine aminotransferase increased  Aspartate aminotransferase increased | Lymphopenia  Neutropenia  Thrombocytopenia  Leukopenia | 1 |  |
| 14 | Anemia  Febrile neutropenia | Lymphopenia  Neutropenia  Thrombocytopenia  Leukopenia | 1 | Glucocorticoid |
| 15 | Anemia  Febrile neutropenia  Neutropenia  Leukopenia | Lymphopenia  Thrombocytopenia | 1 | Glucocorticoid |
| 16 | Febrile neutropenia | Lymphopenia  Neutropenia  Leukopenia | 1 | Glucocorticoid  Plasma exchange |
| 17 | Febrile neutropenia  Lung infection | Heart failure  Lymphopenia  Neutropenia | 3 | Glucocorticoid  Plasma exchange |
| 18 | Anemia  Febrile neutropenia | Heart failure  Lymphopenia  Neutropenia  Thrombocytopenia  Leukopenia | 3 |  |
| 19 | Anemia  Febrile neutropenia  Fever  Thrombocytopenia | Blood bilirubin increased  Lymphopenia  Neutropenia  Leukopenia | 1 | Glucocorticoid |
| 20 | Anemia  Febrile neutropenia  Fever | Lymphopenia  Neutropenia  Thrombocytopenia  Leukopenia | 2 | Glucocorticoid  Plasma exchange |
| 21 | Anemia  Febrile neutropenia | Lymphopenia  Neutropenia  Thrombocytopenia  Leukopenia | 1 |  |
| 22 | Anemia  Febrile neutropenia  Atrial fibrillation  Fever  Creatinine increased | Lymphopenia  Neutropenia  Thrombocytopenia  Leukopenia | 3 | Glucocorticoid  Plasma exchange  Renal replacement therapy |
| 23 | Anemia  Febrile neutropenia | Lymphopenia  Neutropenia  Thrombocytopenia  Leukopenia | 1 |  |
| 24 | Anemia  Febrile neutropenia | Lymphopenia  Neutropenia  Thrombocytopenia  Leukopenia | 3 | Glucocorticoid  Renal replacement therapy |
| 25 | Anemia  Febrile neutropenia | Lymphopenia  Neutropenia  Thrombocytopenia  Leukopenia | 1 |  |
| 26 | Febrile neutropenia | Lymphopenia  Neutropenia  Leukopenia | 1 |  |
| 27 | Febrile neutropenia | Heart failure  Lymphopenia  Thrombocytopenia  Neutropenia  Leukopenia | 3 | Glucocorticoid  Plasma exchange  Renal replacement therapy |
| 28 | Anemia  Febrile neutropenia | Lymphopenia  Neutropenia  Thrombocytopenia  Leukopenia | 1 |  |
| 29 | Anemia  Febrile neutropenia | Lymphopenia  Neutropenia  Thrombocytopenia  Leukopenia | 1 |  |
| 30 | Febrile neutropenia | Lymphopenia  Neutropenia  Leukopenia | 1 |  |

Supplemental Table 6: Adverse Events of the hematological toxicities for patients receiving **anti-BCMA CAR T cells therapy** at three months

| **Adverse event** | **Grade1** | **Grade 2** | **Grade 3** | **Grade 4** | **Grade 3-4** |
| --- | --- | --- | --- | --- | --- |
|  | Number of patients (percent) | | | | |
| Leukopenia | 3 (12.5) | 3 (12.5) | 1 (4.2) | 0 (0.0) | 1 (4.2) |
| Neutropenia | 5 (20.8) | 2 (8.3) | 4 (16.7) | 0 (0.0) | 4 (16.7) |
| Lymphopenia | 4 (16.7) | 1 (4.2) | 2 (8.3) | 0 (0.0) | 2 (8.3) |
| Anemia | 7 (29.2) | 2 (8.3) | 6 (25.0) | 0 (0.0) | 6 (25.0) |
| Thrombocytopenia | 4 (16.7) | 4 (16.7) | 1 (4.2) | 3 (12.5) | 4 (16.7) |

Supplemental Table 7: **The response status for patients receiving anti-BCMA CAR T cells therapy at three months**

| **Myeloma Respons** | **n (Total=30)** | **% (n/30)** |
| --- | --- | --- |
| CR | 5 | 16.70 % |
| VGPR | 5 | 16.70 % |
| PR | 9 | 30.0 % |
| SD | 2 | 6.7 % |
| PD | 6 | 20.0 % |
| Death | 3 | 10.0 % |
| ORR |  | 63.3 % |

CR: complete response; VGPR: very good partial response; PR: partial response;

SD: stable disease; PD: progressive disease. ORR: Objective response rate

Supplemental Table 8: **Comparison of** Mean **fluorescence intensity of** BCMA expression **between baseline and relapse**

| **Patient No.** | Mean **fluorescence intensity of** BCMA expression | |
| --- | --- | --- |
|  | Baseline | Relapse |
| 1 | NE | 227 |
| 6 | 3825 | 1696 |
| 14 | 3137 | 3148 |
| 19 | 2092 | 1656 |
| 22 | NE | 1030 |
| 29 | 1489 | 9503 |
| 30 | 1586 | 1883 |

NE, The expression of BCMA was not evaluated by the multi-parametric flow cytometry but by the validating immunohistochemistry.

**Supplemental figures**


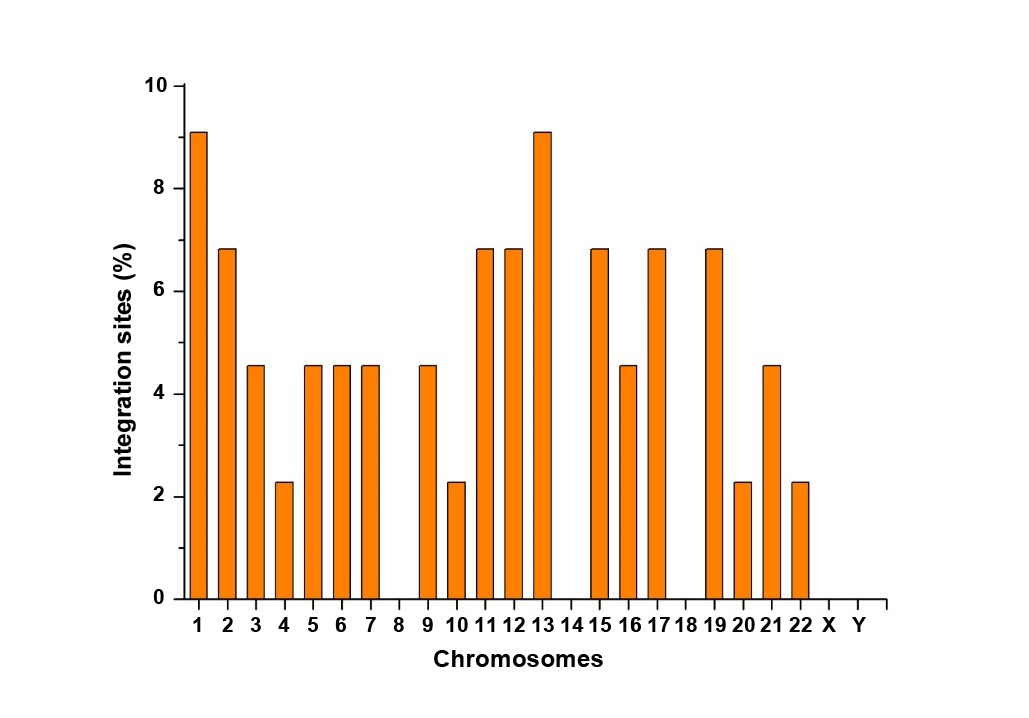


**Figure S1.** Chromosomal distribution of LV integrants in anti-BCMA CAR-T cells. The identification of viral vector flanking genomic sequences is performed with linear amplification mediated PCR (LAM-PCR) ^3^. The human chromosomes are displayed by number. The figure shows the relative fraction of the 45 nrLAM-PCR-identified unique integration sites on each chromosome.


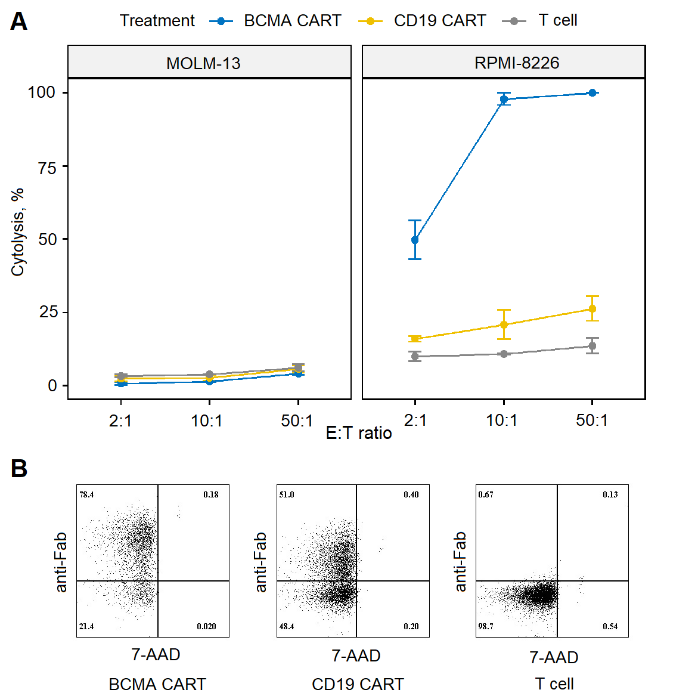


**Figure S2. Anti-BCMA CAR T cells exhibited antileukemic effects in vitro.** Patients underwent leukapheresis to obtain T cells, then the T cells were transduced with lentivirus to express anti-BCMA CAR or anti-CD19 CAR-T according to previously described^2^. (A) Cytolysis-inducing effects of anti-BCMA CAR-T, anti-CD19 CAR-T, and T cells against the MOLM-13 (BCMA negative) and RPMI-8226 (BCMA positive) cell lines were investigated in vitro, at three dose levels of 2:1, 10:1, and 50:1 (Effector to target ratio, E:T ratio). Upper left, the MOLM-13 cells were almost irresponsive to all types of treatment at all dose levels. Upper right, anti-BCMA CAR-T cells showed the strongest cytolysis-inducing effects against RPMI-8226 cells, and a dose-response relationship was observed; by contrast, the effects of anti-CD19 CAR-T and T cells on RPMI-8226 cells were both limited. (B) T cells transfected with anti-CD19 CAR and anti-BCMA CAR were stained with goat anti-mouse-F (ab) 2 antibodies (anti-Fab, Jackson ImmunoResearch), as well as 7-AAD to distinguish alive cells. The transduction rates of anti-CD19 CAR-T and anti-BCMA CAR-T cells were 51.0% and 78.4%, respectively. Shown one representative result of three independent experiments.


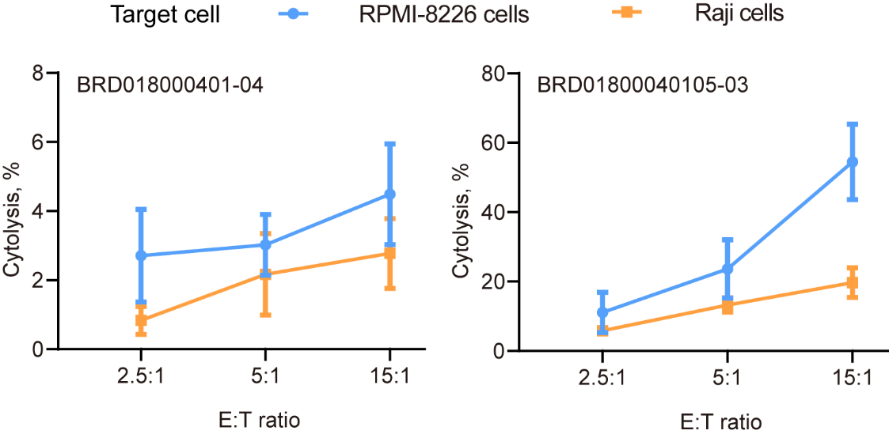


A

B

Day -27

U256 cells i.v.

2x10^6^/mouse

Day -7

Day 0

Day 3

Day 7

NSG: 6-10

week old

Living imaging

CAR-T cells (i.v.)

5x10^6^/mouse

Day 21

Day -14

C


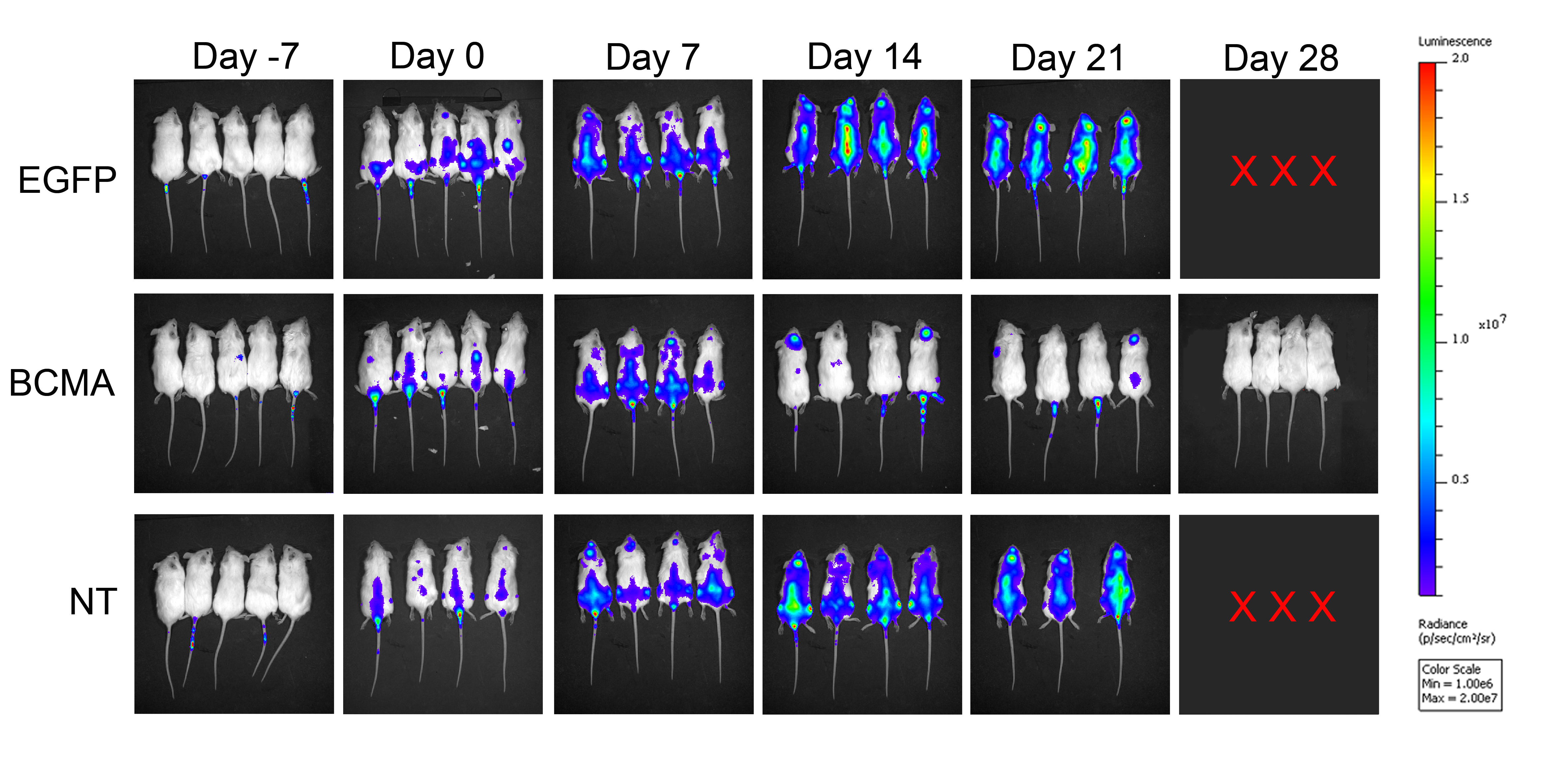


Day -14

Day -7

Day 0

Day 3

Day 7

Day 21


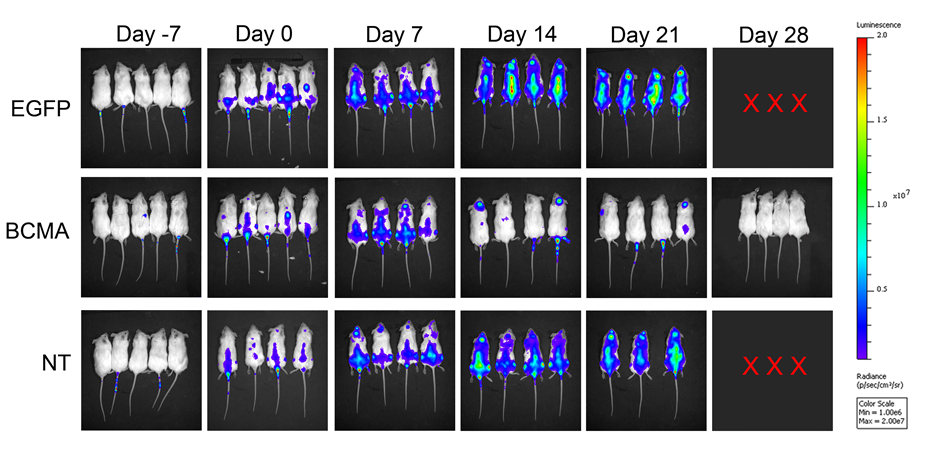

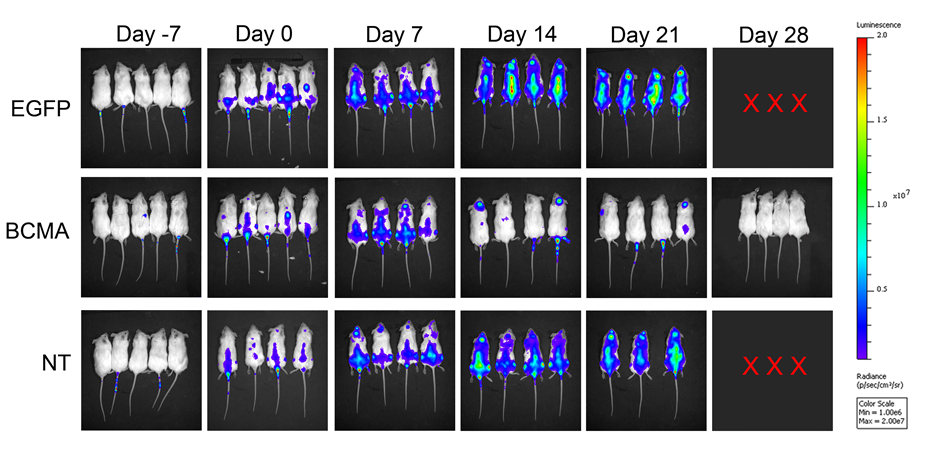


EGFP

BCMA

NT

**Figure S3. Anti-BCMA CAR T cells eradiated U266 cells in a xenograft mouse model.** (A) Cytolysis-inducing effects of anti-EGFP CAR-T and anti-BCMA CAR-T cells against the Raji (BCMA negative) and RPMI-8226 (BCMA positive) cell lines were investigated in vitro, at effector-to-target (E:T) ratios of 2.5:1, 5:1, and 15:1. Anti-BCMA CAR-T cells showed stronger cytolysis-inducing effects than anti-EGFP CAR-T cells against RPMI-8226 cells, and a dose-response relationship was observed; The transduction rates of anti-EGFP CAR-T and anti-BCMA CAR-T cells were 48.9% and 60.3%, respectively. CAR-T cells were manufactured with healthy T cell donors (BRD018000401-04, BRD01800040105-03). (B) The schema for the animal experiments. SCID-gamma chain knockout (NSG) mice were injected with 2 × 10^6^, BCMA positive, luciferase-expressing U266 cells on Day -27. Four weeks after the inoculation, 5×10^6^ anti-BCMA CAR-T cells, non-transduced control T-cell (NT), or anti-EGFP CAR-T cells were infused into the tail vein. These mice were followed with serial weekly imaging to assess the tumor burden of U266 cells. (C) Representative images of leukemia burden by bioluminescent imaging (BLI). Tumors began to be eradicated from days 3 to 21 in the BCMA CAR-T group. *Significant difference (P < 0.05) between the anti-BCMA CAR T cells group and the other two groups.

**
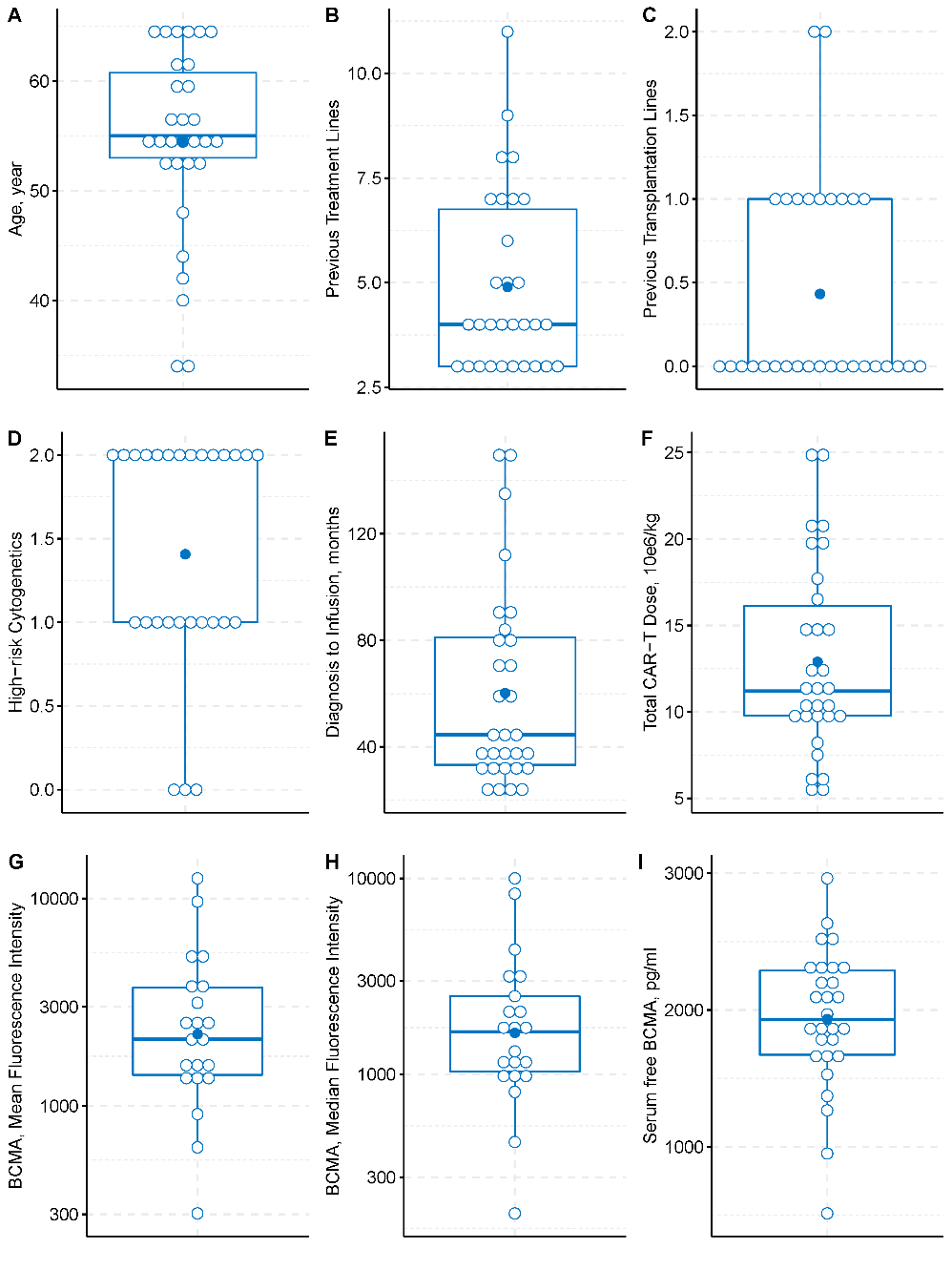
**

**Figure S4.** **Boxplots with binned dots showing the distribution of investigated continuous variables in 30 patients.** (A) Age, year (n=30). (B) Number of previous treatment lines (n=30). (C) Number of previous stem cell transplantation treatment (n=30). (D) Number of high-risk cytogenetic alterations (n=26). (E) Time from diagnosis to the first infusion of CAR T-cells, month (n=30). (F) Total CAR-T dose, 10^6^ cells/kg (n=30). (G) Mean fluorescence intensity of the BCMA (n=20). (H) Median fluorescence intensity of the BCMA (n=20). (I) Serum-free BCMA, pg/ml (n=28). The open circles represent patients’ measurements and the solid circles represent the means.

**
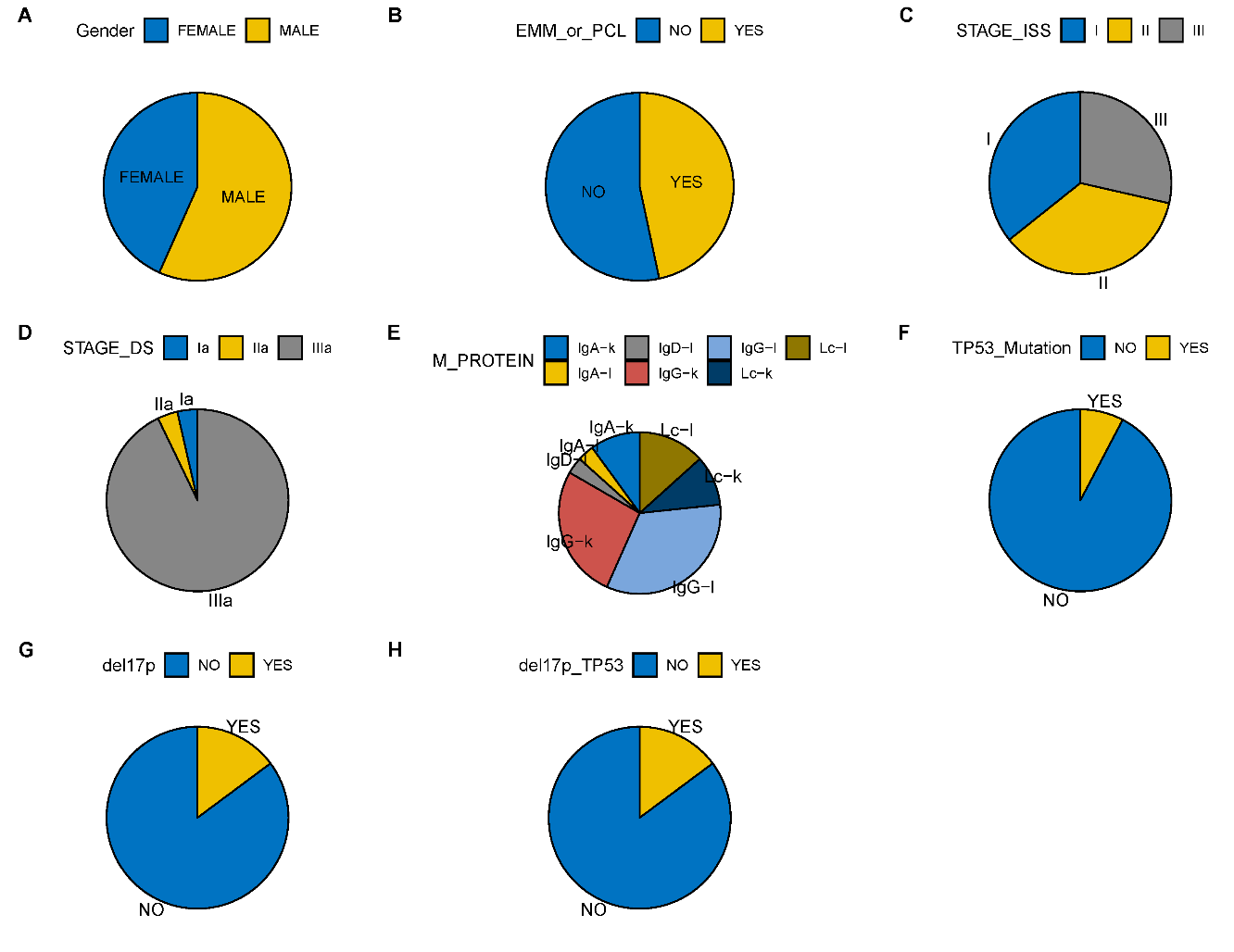
**

**Figure S5. Pie charts showing the distribution of categorical variables investigated in 30 patients.** (A) Gender. (B) Malignancy type; EMM, extra-medullary multiple myeloma; PCL, plasma cell leukemia. (C) Stage by International Staging System, ISS. (D) Durie-Salmon stage. (E) M protein type; Ig, immunoglobulin; Lc, light chain; κ for kappa, and λ for lambda. (F) TP53 mutation measured by next-generation sequencing. (G) del17p measured by interphase fluorescence *in situ* hybridization. (H) Presence of either del17p or TP53 mutation.

**
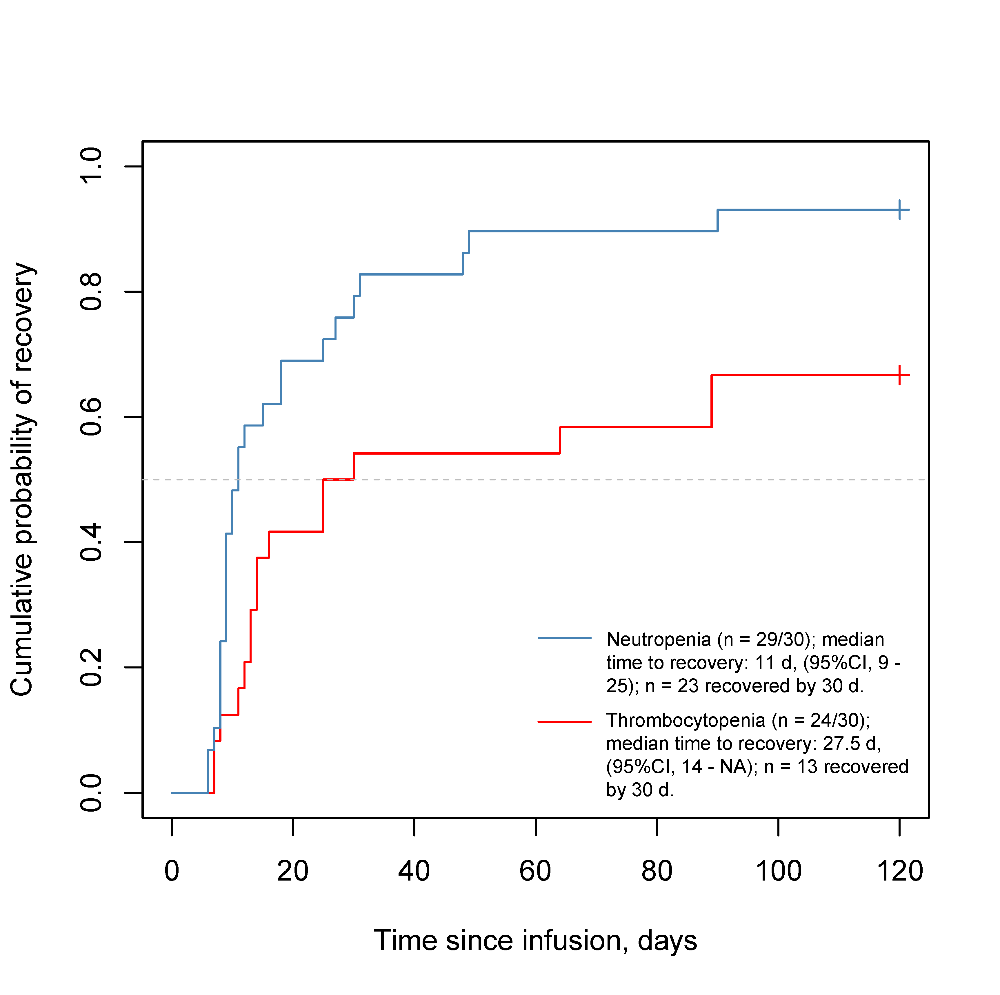
**

**Figure S6.** **Time to Recovery of Grade 3/4 Cytopenias.** Patients with grade 3/4 cytopenia (absolute neutrophil counts < 1.0 ×10^9^/L or platelets < 50 ×10^9^/L based on laboratory values) on or before month one is included. Recovery is defined as absolute neutrophil counts ≥ 1.0 ×10^9^/L and platelets ≥ 50 ×10^9^/L. Time to recovery is defined as the time from infusion to the first time when recovery criteria were met. Median and 95% CI is from Kaplan-Meier estimates.

**
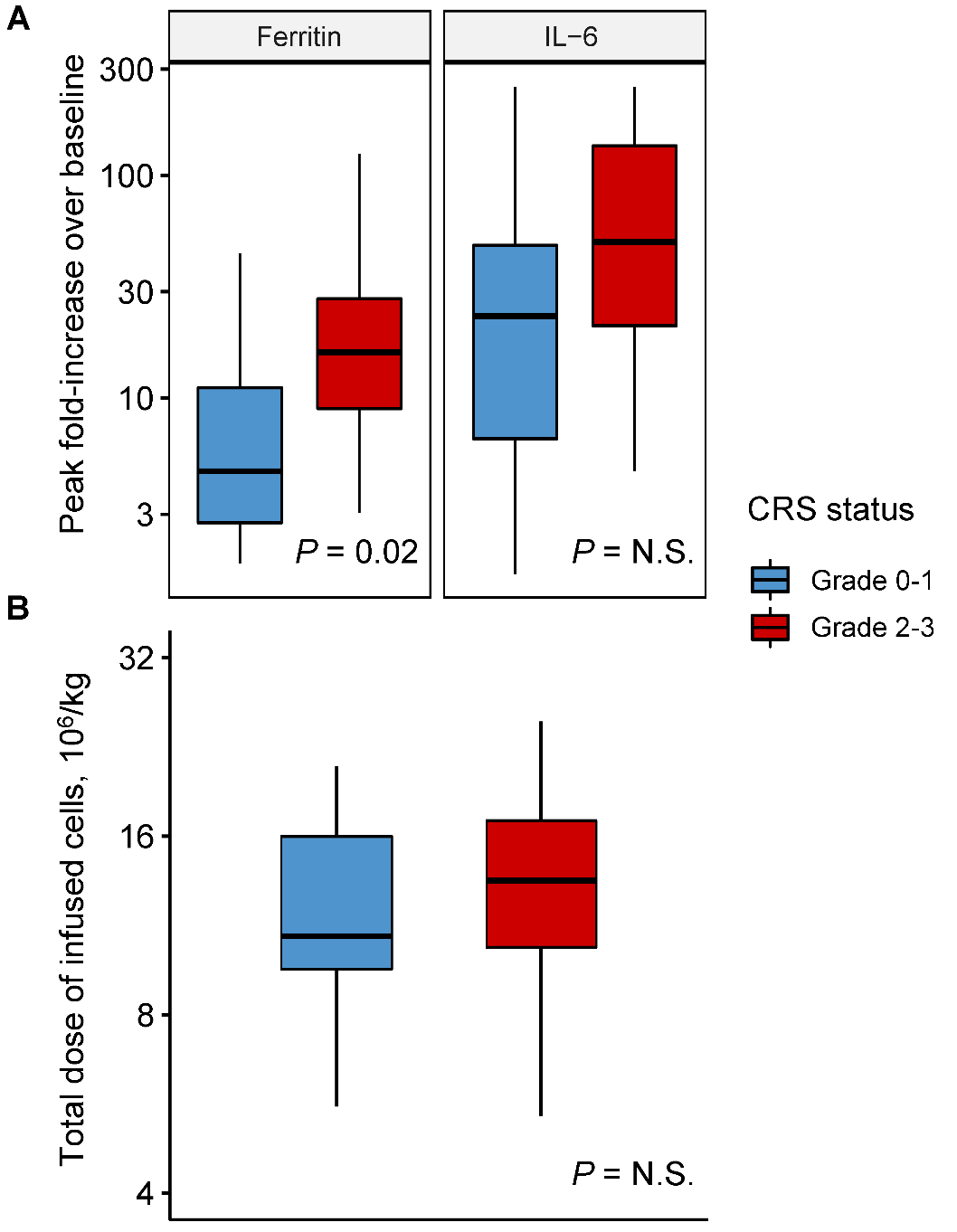
**

**Figure S7. (A) Peak fold-increase over baseline of ferritin (left) and IL-6 (right) levels by cytokine release syndrome severity (CRS).** Grade 0-1, patients free of CRS or with Grade 1 CRS. Grade 2-3, patients with Grade 2 or 3 CRS. A significantly higher increase in ferritin was observed in Grade 0-1 patients compared to Grade 2-3 patients, and a non-significant trend was observed for IL-6. **(B) Infused cell dose by CRS status.** Non-significant association between treatment dose and CRS. *P* values by Wilcoxon rank sum test.


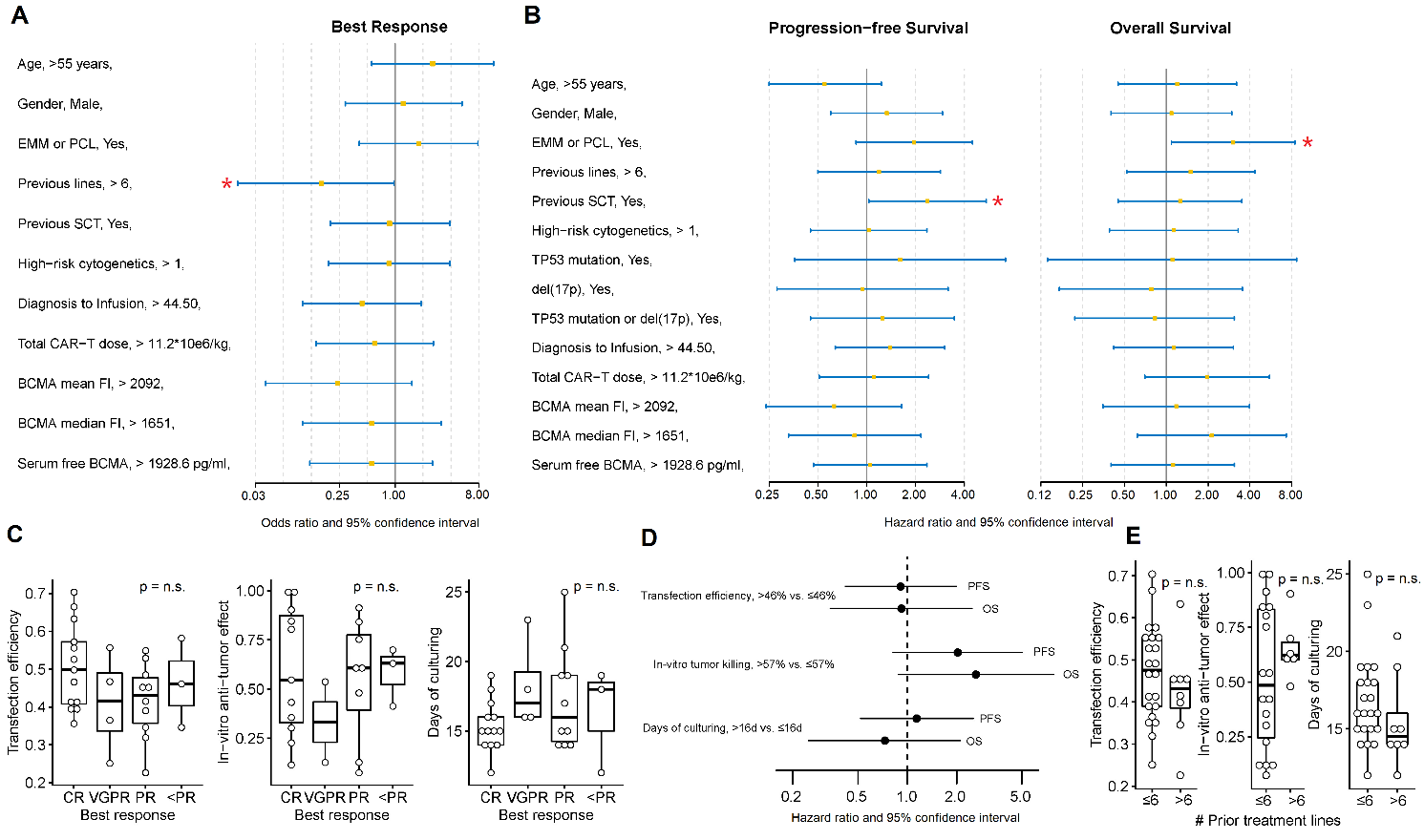


**Figure S8. Forest plots showing the association between factors and efficacy outcomes.** (A) Best response achieved (logistic regression, odds ratio, and 95% confidence interval (95% CI)). (B) Progression-free survival (Cox regression, the hazard ratio (HR) and 95% CI; left), and overall survival (Cox regression, HR, and 95% CI; right), respectively. As highlighted with red “*” number of previous treatment lines (>6 vs. <=6), prior stem cell transplantation (yes vs. no), and presence of extra-medullary disease or plasma cell leukemia (yes vs. no) was the only significant factor associated with the best response, progression-free survival, and overall survival, respectively (*P* < 0.05). (C) Association between CAR T manufacturing characteristics (transduction efficiency, in-vitro tumor-killing effect, and days of culturing) and patients’ response (CR, VGPR, PR, and < PR). (D) Association between CAR T manufacturing characteristics and patients’ prognosis (PFS and OS). (E) Association between CAR T manufacturing characteristics and patients’ number of prior treatment lines. Abbreviations: CR, complete response; VGPR, very good partial response; PR, partial response.


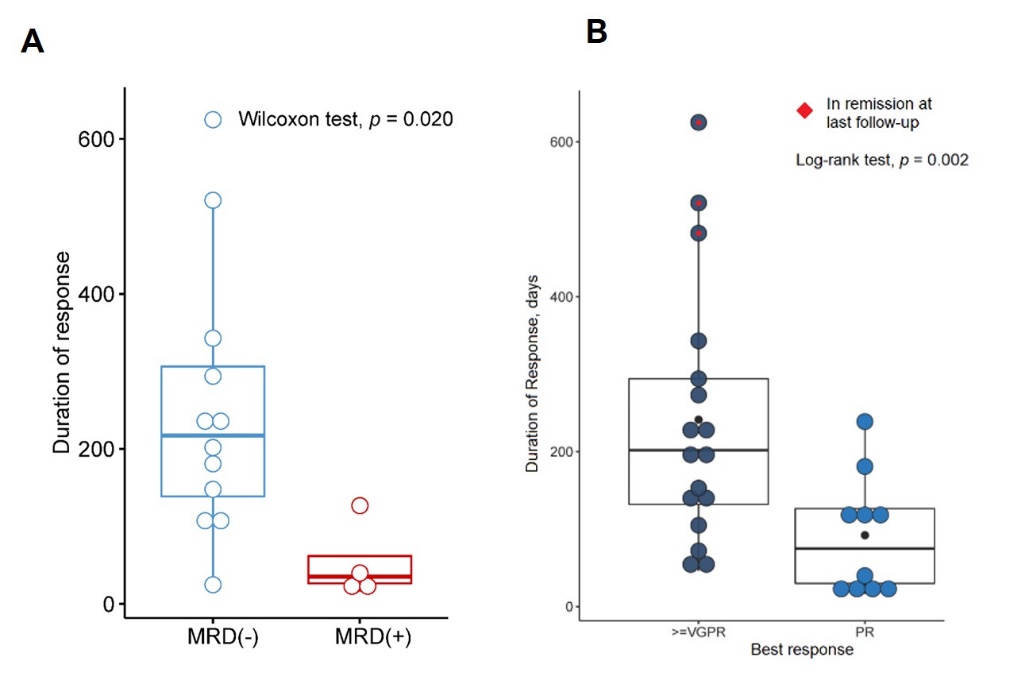


**Figure S9.** (A) The DOR (duration of response) of four MRD-positive patients who achieved PR was significantly shorter than that of 12 MRD-negative patients who achieved PR or better. (Median, 35 vs. 217.5 days, Wilcoxon rank-sum test, p = 0.020). MRD (+), MRD-positive; MRD (-), MRD-negative. (B) Duration of response between patients with ≥VGPR response and others. ≥VGPR responders had a significantly longer duration of response than others.


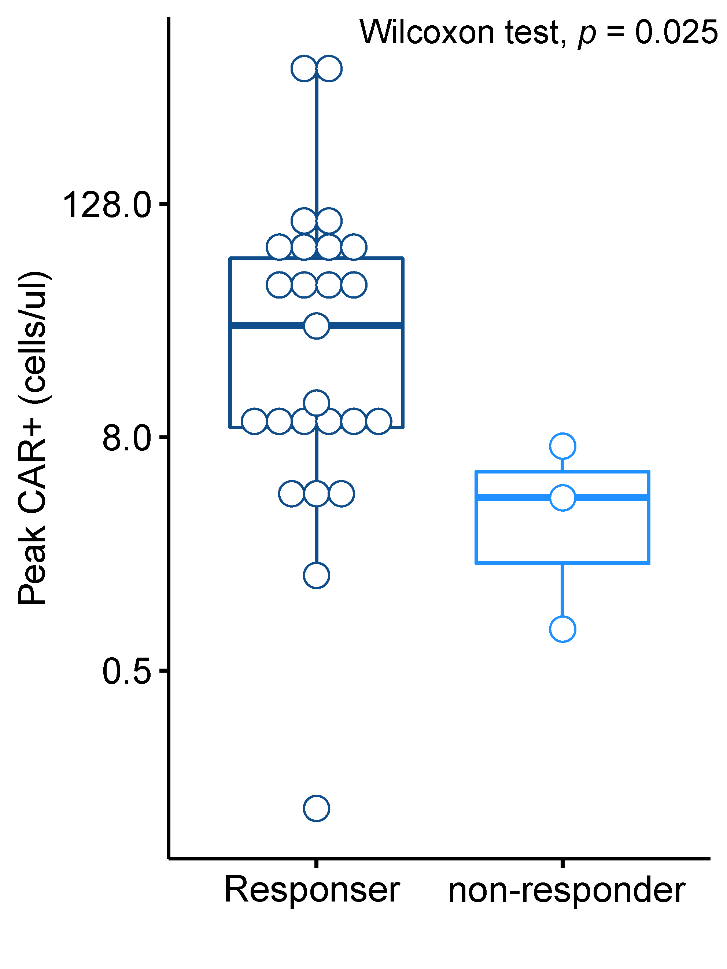


**Figure S10.** **Peak blood CAR^+^ cell levels were higher in responders versus nonresponders. (p = 0.025, Wilcoxon rank-sum test; n = 25 versus 3).** The percentage of peripheral blood lymphocyte cell that was CAR^+^ cells was determined by Multiparameter flow cytometry. The absolute number of CAR^+^ cells/mL of blood was determined by multiplying the percentage of CAR^+^ cells by the sum of blood lymphocytes/mL.

**
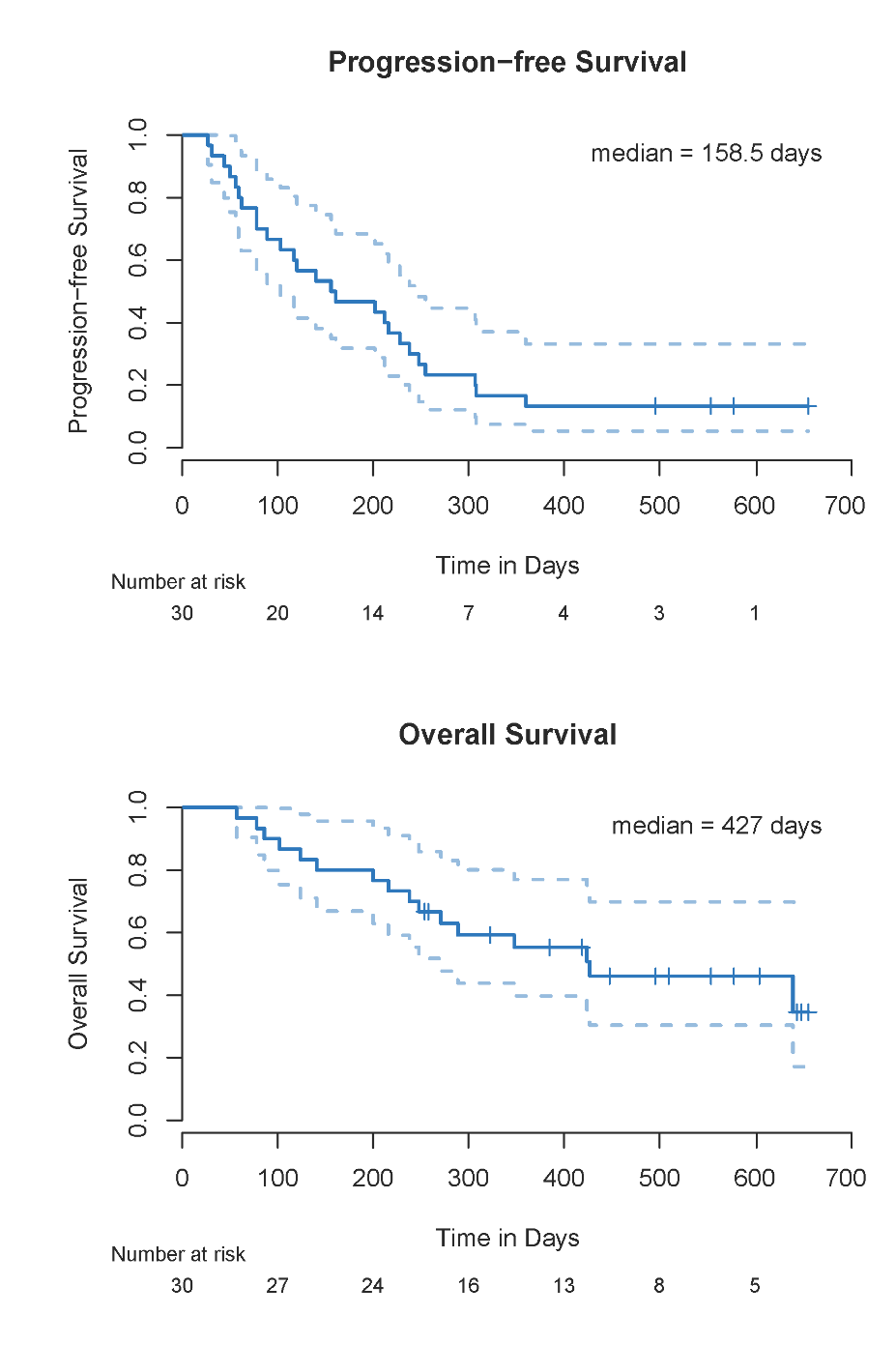
**

**Figure S11.** **Long-term outcome of 30 patients.** Progression-free survival (Upper) and overall survival (Lower).


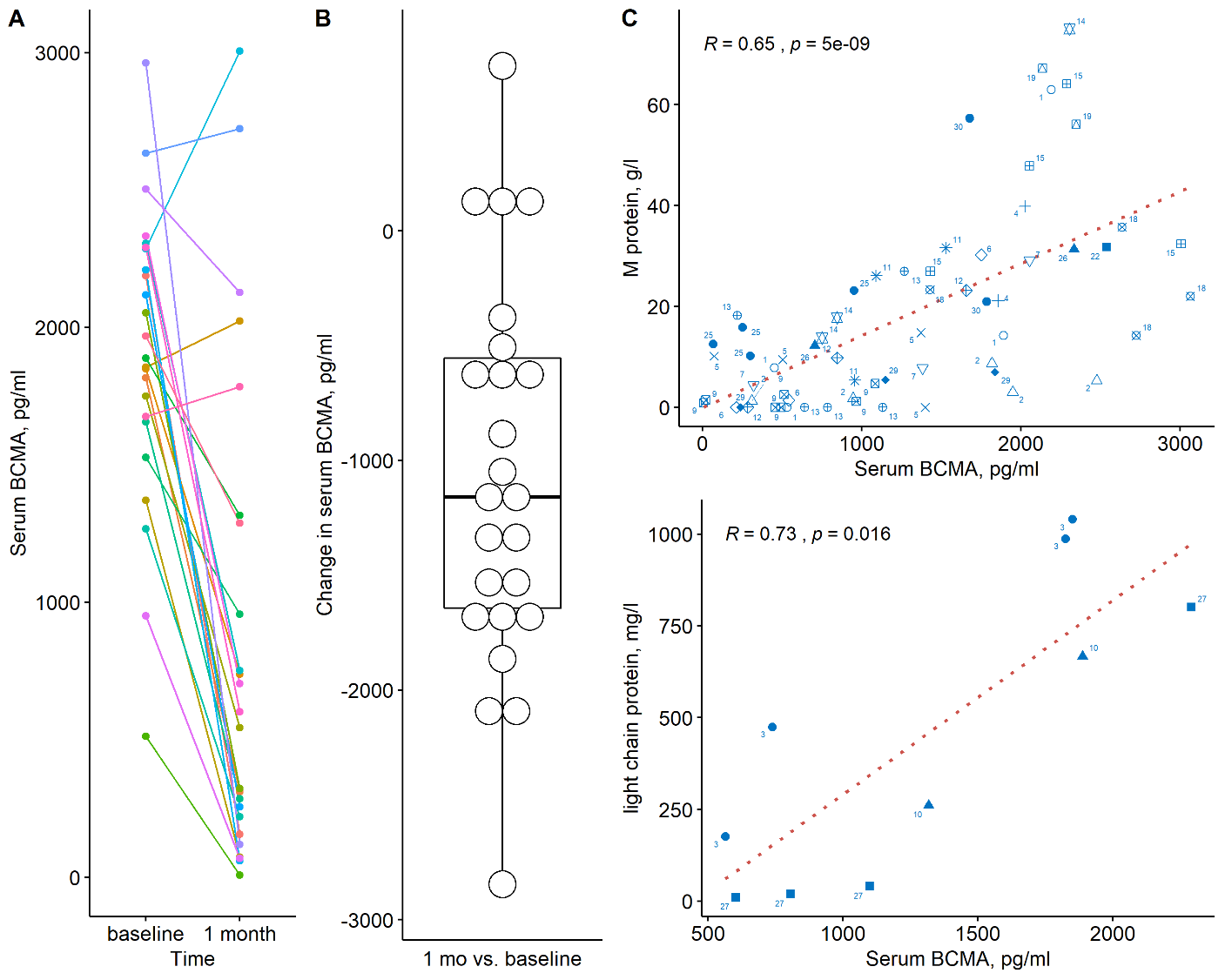


**Figure S12. Serum BCMA level dynamics and its correlation with M proteins.** (A) Baseline and 1-mo post-infusion serum BCMA level in 30 patients. (B) Distribution of difference 1-mo vs. baseline BCMA level across 30 patients. Significant reduction in the serum BCMA level was observed (Wilcoxon signed-rank test, P < 0.01). (C) Correlation between serum BCMA and M-protein (Upper) or light chain protein (Lower) in the patients. Data of different patients were indicated with different symbols and patient ID numbers. Significant positive correlations revealed (P < 0.05).

**References**

1. Xu H, Wang N, Cao W, et al. Influence of various medium environment to in vitro human T cell culture. *In Vitro Cell Dev Biol Anim* 2018; 54:559-566.

2. Xu H, Cao W, Huang L, et al. Effects of cryopreservation on chimeric antigen receptor T cell functions. *Cryobiology* 2018; 83:40-47.

3. Paruzynski A, Arens A, Gabriel R, et al. Genome-wide high-throughput integrome analyses by nrLAM-PCR and next-generation sequencing. *Nat Protoc* 2010; 5:1379-95.

.
