# Supplementary material for: A phase I study of anti‐BCMA CAR T cell therapy in relapsed/refractory multiple myeloma and plasma cell leukemia
Source: Clin Transl Med. 2021 Mar 9;11(3):e346. doi: 10.1002/ctm2.346 (PMC7943908; doi:10.1002/ctm2.346)
Supplement: Supplementary file 2 — Protocol [file CTM2-11-e346-s001.docx]

**Protocol**This trial protocol has been provided by the authors to give readers additional information about their work.
Chunrui Li, Wenyue Cao, Qiuxiang Wang, et al. **A phase I study of anti-BCMA CAR T cell therapy in** **relapsed/refractory multiple myeloma and plasma cell leukemia**

.

**This supplement contains the following items:**

- 1. Selection of patients, including both eligibility and ineligibility criteria
  2. Schema and treatment plan, including administration schedule
  3. Rules for dose modification
  4. Measurement of treatment effect, including response criteria, definitions of response and survival, and methods of measurement
  5. Reasons for early cessation of trial therapy
  6. Objectives and entire statistical section (including endpoints)

**Clinical study protocol**

An open-label, single-center, and single-arm pilot trial of anti-BCMA CAR T cells therapy for R/R BCMA-positive plasma cell neoplasms.

Study Sponsor: Wuhan Bio - Raid Biotechnology Co., Ltd.  
Protocol Version: Version 3.0

1. **Selection of patients, including both eligibility and ineligibility criteria**

**1.1 Study population**

This study will enroll subjects with BCMA+ neoplasms.

**1.2 Number of Subjects**

30~50 evaluable subjects will be enrolled.

**1.3 Eligibility**

**Inclusion Criteria**

1. Voluntarily sign an informed consent form(s) and ability and willingness to adhere to the study visit schedule and all protocol requirements
2. 18~70 years of age at the time of signing an informed consent
3. Evidence of cell membrane BCMA expression by either multiparameter flow cytometry (MFC) or immunohistochemistry (IHC) of formalin-fixed paraffin-embedded tumor tissue (e.g., bone marrow plasmacytoma biopsies). In this study, BCMA+ neoplasms were defined as conditions with at least one of the following characteristics: 1) BCMA expression detected in ≥ 50% of malignant plasma cells by IHC; 2) BCMA expression detected in ≥ 20% of malignant plasma cells from fresh marrow aspirates by MFC, and 3) Mean fluorescence intensity (MFI) of BCMA expression ≥ 1000 as determined by MFC. Relapsed or refractory to previous treatments, including hematopoietic stem cell transplantation, or voluntarily to accept anti-BCMA CAR-T cell therapy as the salvage therapy.
4. BCMA+ neoplasms included: Leukemia; Lymphoma; Multiple myeloma
5. Subjects must meet the following criteria:

- Subjects must have the residual disease after primary therapy, but not eligible for hematopoietic stem cell transplantation
- Relapse after complete response, but not eligible for hematopoietic stem cell transplantation
- Have high-risk factors
- Recurrence or no remission after hematopoietic stem cell transplantation or cellular immunotherapy

1. Subjects must have measurable disease
2. Essentially adequate organ function is necessary for eligibility. 1) The adequate hepatic function defined by aspartate aminotransferase (AST) and/or alanine aminotransferase (ALT) ＜ 3.0 $\times$ upper limit of normal (ULN) and total bilirubin ≤ 34.2µmol/L; 2) Adequate renal function defined by serum creatinine ＜220. 0 µmol/L;3) Lung function: indoor oxygen saturation ≥ 95%; 4) Left ventricular ejection fraction ≥ 40%
3. Without a history of accepting anti-cancer therapy, including chemotherapy, radiotherapy, immunotherapy (immune-suppressive drug treatment) within four weeks of screening
4. Adequate venous access for apheresis
5. Measurable diseases, Eastern Cooperative Oncology Group performance status 0~2, and a life expectancy of three months or more are necessary for eligibility

**Exclusion Criteria**

1. Pregnant or lactating women
2. Planning pregnancy within one year
3. Subjects cannot guarantee effective contraception within one year after enrollment
4. Presence of uncontrollable infection within four weeks before the start of conditioning
5. Active hepatitis B, active hepatitis C infection at the time of screening
6. Known human immunodeficiency virus (HIV) positivity
7. Suffering from an immunodeficiency disease or severe autoimmune disease
8. The patient is allergic to macromolecular Biopharmaceuticals such as antibodies or cytokines
9. Investigational other clinical research within six weeks before the start of conditioning
10. Receiving systemic steroid therapy within four weeks before the start of conditioning (Except for patients with inhaled hormones)
11. Suffering from mental illness
12. Having drug abuse/addiction
13. The patient had other conditions, as assessed by the investigator, that was not suitable for a clinical study
14. **Schema and treatment plan, including administration schedule**

**2.1** **Lymphodepletion Treatment Plan**

All eligible patients underwent leukapheresis. A lymphodepleting chemotherapy with cyclophosphamide 20mg/kg and fludarabine 25 mg/m^2^ daily on days -4 to -2 was administered before infusion of CAR-BCMA T cells on day 0. Related laboratory and imaging evaluations were performed for toxicity and response assessment according to the study procedures. According to local institutional guidelines, anti-emetics may be administered, but dexamethasone or other steroids are not to be administered.

CAR-BCMA T cells must be delivered to the subject care unit and infused immediately within 1 hour; all procedures involving CAR-BCMA T cells must be performed using aseptic techniques by trained personnel.

**2.2 Cytokine Release Syndrome (CRS) Definition and Management**

This protocol will follow the recommendations and management for CRS, as defined by Lee et al. (2014).

The work-up of a CRS should include hospitalization and evaluation for an infectious etiology (e.g., blood cultures, urine culture, chest X-ray, as required). During the days after infusion, it is recommended that subjects must be hospitalized until the symptoms of CRS disappear, and the blood routine reaches the discharge standard. Because of the risk of CRS, subjects treated on this protocol must be hospitalized until the symptoms of CRS disappear, and the blood routine reaches the discharge standard.

**2.3 Patients follow-up**

All patients were followed till death, loss to follow-up, or withdrawal of consent. All subjects who complete the study will be asked to continue to undergo long-term follow-up for up to 15 years after their last infusion, with a focus on long-term safety and efficacy. Detailed data regarding general information on enrollment, clinical manifestation at diagnosis, treatment, and follow-up were collected. Laboratory data recorded included results of routine medical work-up, peripheral blood (PB) and bone marrow (BM) examination, immunophenotyping, cytogenetic and genomic aberrations (karyotyping, multiplex fusion gene testing, fluorescence *in-situ* hybridization, and next-generation targeted exome sequencing), diagnostic imaging and copy number changes of CAR T-cell, and minimal residual disease (MRD) screening (Multiparameter flow cytometry or IHC).

Duration of response was defined as the time from the first objective partial response or better to the first disease progression or death due to any cause, whichever occurred first. Progression-free survival was defined as the time from the first anti-BCMA CAR T Cells infusion to the occurrence of either the first disease progression or death due to any cause. Time to recovery of grade 3/4 cytopenia was defined as the time from the occurrence of the first grade 3/4 event to the first date of a stable recovery. All the above mentioned time-to-event outcomes were measured in days.

**Schedule of events screening, anti-BCMA CAR T Cells infusion, and follow-up**

| Procedure | Screening period  D (day) | Pretreatment chemotherapy period  D (day) | | Short-term follow-up period  D (day) | | | | | | | The medium and long term follow-up period  D (day) | | | Last study visits  D (day) |
| --- | --- | --- | --- | --- | --- | --- | --- | --- | --- | --- | --- | --- | --- | --- |
|  | D-21 | D (-4 to -2) | D-1 | D0 | D30 | D60 | D90 | D120 | D150 | D180 | D240 | D300 | D360 | Anytime |
|  |  |  |  |  | ± 5 | ± 5 | ± 5 | ± 5 | ± 5 | ± 5 | ± 7 | ± 7 | ± 7 |  |
| Physical examination | √ | √ | √ | √ | √ | √ | √ | √ | √ | √ | √ | √ | √ |  |
| Neurological evaluation | √ | √ | √ | √ | √ | √ | √ | √ | √ | √ | √ | √ | √ | √ |
| Blood for serum pregnancy | √ |  |  |  |  |  |  |  |  |  |  |  |  | √ |
| Blood for clinical laboratory tests* | √ |  |  | √ | √ | √ | √ | √ | √ | √ | √ | √ | √ | √ |
| Electrocardiogram | √ |  |  | √ | √ |  | √ |  | √ |  | √ | √ | √ | √ |
| Blood Soluble BCMA | √ |  |  |  | √ | √ | √ | √ | √ | √ | √ | √ | √ | √ |
| EBV copy in peripheral blood | √ |  |  |  | √ |  | √ |  | √ |  | √ | √ | √ | √ |
| Lentivirus copy (qPCR) | √ |  |  |  | √ | √ | √ | √ | √ | √ | √ | √ | √ | √ |
| Bone marrow biopsy/aspirate^@^ | √ |  |  |  | √ | √ | √ | √ | √ | √ | √ | √ | √ | √ |
| Morphology/BCMA+ cells/Cytogenetics/FISH | √ |  |  |  |  |  |  |  |  |  |  |  |  |  |
| Minimal Residual Disease ^&^ |  |  |  |  | √ | √ | √ | √ | √ | √ |  |  |  | √ |
| Blood CAR+ T cells |  |  |  |  | √ | √ | √ | √ | √ | √ | √ | √ | √ | √ |
| IL-6, Ferritin, and C-reactive protein | √ |  |  | √ | √ | √ | √ | √ | √ | √ |  |  |  | √ |
| Lymphodepletion |  | √ |  |  |  |  |  |  |  |  |  |  |  | √ |
| CAR-T cell infusion |  |  |  | √ |  |  |  |  |  |  |  |  |  | √ |
| Adverse Event collection | √ | √ | √ | √ | √ | √ | √ | √ | √ | √ | √ | √ | √ | √ |
| Clinical Disease Staging/Response Assessment^#^ | **√** |  |  |  | √ | √ | √ | √ | √ | √ | √ | √ | √ | √ |

*Clinical laboratory tests are to be performed by the local laboratory and include the following: the complete blood count, liver function, renal function, Serum beta-2-microglobulin, and coagulation.

^@^Bone Marrow Biopsy and Aspirate: Bone Marrow Biopsy and Aspirate will be performed according to the protocol and may be done in suspected progressive disease as applicable.

^&^Minimal Residual Disease aspirate for assessment will be provided for assessment only at suspected CR

^#^MM response assessments include the following:

- Serum and urine immunofixation, Serum Free Light Chain (FLC, kappa and lambda), Quantification of Ig (IgG, IgM, IgA), Serum (SPEP) and urine (24-hour collection) (UPEP) electrophoresis for M-protein measurement.
- Skeletal Survey: At baseline and at any time post cell infusion if the treating investigator believes there are signs or symptoms of increased or new skeletal lesions.
- Radiographic Disease Assessment: Should be performed in any subjects with the documented extramedullary disease, according to the schedule of assessments. The same imaging modality used for screening (MRI, PET, CAT, or PET/CAT) should be used throughout the study.

1. **Rules for dose modification**

Subjects may receive 5.0 – 30.0 × 10^6^ CAR+ cells/kg according to the number of anti-BCMA CAR T Cells manufactured. Subjects with a number of CAR+ T cells manufactured below 5.0 × 10^6^ CAR+ cells/kg were excluded from this study.

1. **Measurement of treatment effect, including response criteria, definitions of response and survival, and methods of measurement**

Response assessments will be made according to the IMWG Uniform Response Criteria for Multiple Myeloma. Response assessments include the following:

- Serum and urine immunofixation, Serum Free Light Chain (FLC, kappa and lambda), Quantification of Ig (IgG, IgM, IgA), Serum (SPEP) and urine (24-hour collection) (UPEP) electrophoresis for M-protein measurement
- Skeletal Survey: At baseline and at any time post cell infusion if the treating investigator believes there are signs or symptoms of increased or new skeletal lesions
- Radiographic Disease Assessment: Should be performed in any subjects with the documented extramedullary disease, according to the schedule of assessments. The same imaging modality used for screening (MRI, PET, CAT, or PET/CAT) should be used throughout the study
- Percent of plasma cells and BCMA expression will be assessed on bone marrow biopsy and aspirate samples collected per Schedule of Events and as clinically indicated to assess response according to the IMWG Uniform accurately
- Bone marrow assessments should include flow cytometry, fluorescence in situ hybridization (FISH), Cytogenetics, and morphology. Bone marrow aspirate will also be used for the evaluation of MRD at appropriate time points. If a bone marrow biopsy or aspirate is performed at any time during the study, biopsy and aspirate samples should be collected in the clinical response assessments and potential research if available

1. **Reasons for early cessation of trial therapy**

5.1 Study Withdrawal

Subjects may withdraw from this study at any time, for any reason. Other than progressive disease or death, other possible reasons for study withdrawal include:

- Toxicity
- Subjects were reluctant to continue participating in the study;
- Subjects can withdraw from the study after subjects experienced severe AE or laboratory abnormalities with significant clinical significance
- Subjects have poor adherence to the protocol
- Failure of transduced cells to be dispositioned for clinical use
- Closure of the study

5.2 Study Pausing Criteria

- Enrollment in this study may be paused at any time for safety reasons
- The research department or the administrative department requested to pause the study
- There are significant errors in the protocol

1. **Objectives and entire statistical section (including endpoints)**

6.1 Study Objectives

The primary objective of the study is to: Confirm the safety of CAR-BCMA T cell therapy for R/R BCMA-positive plasma cell neoplasms

The secondary objective of the study is to:

- Provide preliminary efficacy data on the anti-tumor effects of treatment with CAR-BCMA T cell therapy in R/R BCMA-positive plasma cell neoplasms
- Evaluate the persistence of CAR-BCMA T cell in the blood

6.2 Study Endpoints

The primary endpoints of the study are safety, including the incidence of adverse events (AEs), severe adverse events (SAE), and cytokine release syndrome (CRS).

The secondary endpoints of the study are:

- The objective response rate (ORR) at three months, six months, and one year.
- Overall survival
- Progression-free survival
- Detection and quantification of CAR-BCMA T cell in blood, bone marrow or tumor tissue over time
  1. **Statistical Methods**

Descriptive summary statistics, as well as 2-sided, 95% confidence intervals will be presented on selected parameters. For categorical variables, summary tabulations of the number and percentage within each category of the parameter will be presented. The analysis of categorical variables was performed using Clopper-Pearson 95% confidence interval (CI) and Fisher’s exact test. For continuous variables, the number of observations, mean, median, standard deviation, minimum and maximum values will be presented. Wilcoxon rank-sum test was applied to continuous variables. Estimates of overall survival and progression-free survival will be presented using Kaplan-Meier curves. These analyses will be provided for the efficacy population. Cox regression was used to assess the association between factors and survival. All treated subjects will be included in the assessment of safety. Adverse events will be coded using the Medical Dictionary for Regulatory Activities (MedDRA). Adverse events will be summarized by the MedDRA system organ class and preferred terms, and separate tabulations also will be produced for related adverse events.
